# Radiolabelled Extracellular Vesicles as Imaging Modalities for Precise Targeted Drug Delivery

**DOI:** 10.3390/pharmaceutics15051426

**Published:** 2023-05-06

**Authors:** Sumel Ashique, Krishnan Anand

**Affiliations:** 1 Department of Pharmaceutics, Bharat Institute of Technology (BIT), School of Pharmacy, Meerut 250103, India; 2 Department of Chemical Pathology, School of Pathology, Faculty of Health Sciences, University of the Free State, Bloemfontein 9300, South Africa

**Keywords:** radiolabelled, extracellular vesicles, imaging, drug delivery, biomedical applications

## Abstract

Extracellular vesicles (ECVs) have been abandoned as bio-inspired drug delivery systems (DDS) in the biomedical field. ECVs have a natural ability to cross over extracellular and intracellular barriers, making them superior to manufactured nanoparticles. Additionally, they have the ability to move beneficial biomolecules among far-flung bodily cells. These advantages and the accomplishment of favorable in vivo results convincingly show the value of ECVs in medication delivery. The usage of ECVs is constantly being improved, as it might be difficult to develop a consistent biochemical strategy that is in line with their useful clinical therapeutic uses. Extracellular vesicles (ECVs) have the potential to enhance the therapy of diseases. Imaging technologies, particularly radiolabelled imaging, have been exploited for non-invasive tracking to better understand their in vivo activity.

## 1. Introduction

According to several studies carried out over the years, different cellular types produce little lipid particles that act as a mirror of the activities that take place inside the cells. Since ECVs are more complex than they first appear to be, they have been divided into groups based on their size, metabolic make-up, and cell of origin [1]. Whatever their origins, the cargo of ECVs has emerged as the most intriguing subject. Among the cargo carried by ECVs are proteins, lipids, nucleic acids such as mRNA, and other bioactive materials. Later research confirmed the crucial role ECVs play in cell-cell communication [2] and their connection to a variety of disorders, such as diabetes [3], inflammatory conditions [4], and cancer [4]. ECVs are essential intermediaries of cell-cell interaction because they carry or deliver actionably active biological molecules, such as proteins, nucleic acids, and lipids in the DNA role, including RNA, into the adjoining tissues or to detached bearer cells, where they lead to a biologic answer [5,6]. According to Maas et al., (2017) [2] intercellular communication via ECVs appears to have a role in the control of a number of physiological processes as well as the pathogenesis of a number of diseases, including inflammation, cancer, neurological, cardiovascular, and autoimmune disorders. In fact, ECVs carry distinctive payloads that reflect the location and affliction of the benefactor cell and may also be applied as biomarkers for scanning for investigations, including prognosis purposes [7,8]. In addition, ECVs may pass through biological barricades, breach impenetrable structural tissues, and move safely in extracellular fluids while distributing endogenous cargo to desired cells with high efficiency and selectivity [5]. Due to these factors, ECVs have attracted a lot of attention as possible drug delivery systems for regenerative medicine, immunomodulation, and anti-tumor therapy [9]. ECVs have advantages over organic or inorganic nanoparticles in that they have inherent biocompatibility, elevate physicochemical resilience, have low immunogenicity, and have long-distance information, including an inherent aiming potential to intermingle along cells through membrane fusion with signal transduction [10,11]. The lack of knowledge of ECVs’ in vivo bio function in real stint, despite efforts and advancements in the field, poses a significant obstacle to their usage in diagnostic or therapeutic settings [12]. For ECVs to be used effectively as a curative or drug deployment framework in bioscience utilizations, it is necessary to determine their circulation kinetics and biodistribution profile, as well as their aiming potency to exact cells or tissues and uptake route, including cargo transport reliability to inheritor cells [13]. Exogenous in vivo comportment of ECVs can be accurately understood using a non-invasive molecular envisioning approach [14,15]. Nuclear imaging stands out among the various preclinical research modalities, mainly because of its excellent profile regarding sensitivity and safety, as well as its huge potential for medical transformation. With attention on nuclear-powered imaging methods, including positron emission tomography (PET) with single-photon emission tomography (SPECT), over and above the state of the art for radiolabelling performances for ECVs, we will make available a general outline of the molecular envisioning practices that are accessible for the tracking of ECVs in vivo in this article.

## 2. Advantages of ECVs in Drug Delivery System

As a nominally aggressive synthetic drug delivery deployment approach has been devised to daze restrictions of the unrestricted therapeutics and overcome diverse biological blockades across patient diseases and populations, tailored clinical interventions are becoming more and more essential for therapeutic efficacy. Nanoparticles have been developed as a delivery method to enhance the issuance or spreading therapeutic model, with a focus on controlling the intervention through the attributes of the vehicle rather than the physicochemical properties of the drug molecule [16]. The use of nanoparticles is still accompanied by a number of disadvantages despite the benefits they provide, such as increasing the solubility and steadiness of encapsulated consignments, encouraging passage across membranes, and delaying circulation times to increase the safety and efficacy for delivery of therapeutics [17]. The target organ dose is notable, represented by the reticuloendothelial system’s quick clearance [18], congregation in the spleen and liver, and immediate hypersensitive reaction [19,20]. Extracellular vesicles (ECVs) are one of the areas of interest that is constantly rising in the field of bio-inspired drug delivery systems, or biologics. Our comprehension of novel types of cell-cell communication has been enhanced by these assorted populations of naturally derived membrane vesicles of nano-to-micro-sized vesicles that can transfer biological molecules from manufacturer to receiver cells [21]. These systems share a significant benefit in that they are derived from living cells, which makes them appealing for commercial product development. Cell-derived ECVs carry a biological payload that modulates immune responses while promoting angiogenesis and tissue repair [22]. This has focused attention, in particular, on using ECVs for therapeutic delivery to get around problems with synthetic drug delivery methods. The circulation, central stability, and capacity of ECVs to deliver and shield a wide range of nucleic acids into inheritor cells while evading the mononuclear phagocytic system (MPS) through displaying the exterior protein CD47 are further noteworthy features of ECVs [23]. Furthermore, it is important to note that ECVs may contain proteins which attach to and organize its RNA [24]. Consequently, ECVs are a prospective source for building systems to deliver therapies in a variety of clinical settings, including in vivo gene editing, immunotherapy, and cancer treatment. In mouse experimental settings, radioisotope labelling of ECVs employing radiotracers with clinical validation and nuclear imaging have both been utilized to track ECVs [25]. These methods allow for precise detection, even in deep organs, but they call for instruments that are not common in research departments. As an alternative, ECVs can be easily labelled with fluorescent dyes; these dyes have been used to mark membrane components and there are also dyes that are selective for the DNA and RNA found in ECVs [26].

## 3. ECVs as Nanomedicines

The potential for ECVs or exosomes (200 nm) to serve as therapeutic nanomedicines has been studied among the many ECV subtypes [27,28]. By more effectively targeting illness locations and/or lowering systemic harmful side effects, nanomedicine seeks to enhance the therapeutic effects of medications [29]. Small extracellular vesicles (SECVs) are a desirable platform for nanomedicine due to a number of factors. Natural ECVs include antigens and, dependent on the cell of origin, may have particular tissue-approaching capabilities in comparison to other synthetic platforms (such as liposomes and polymers) [30,31]. As drug delivery vehicles [32], SECVs can be correspondingly fabricated to exhibit ligands for improved cell or tissue aiming, including steadiness. They have also been shown to accumulate in tumors as a result of the increased permeability and retention (EPR) effect [33]. Their natural capacity to traverse the blood-brain barrier (BBB) i.e., a considerable biological barrier for various other nanomedicine drug delivery (DD) platforms, is another key characteristic that is frequently mentioned [34]. However, there is currently a dearth of knowledge surrounding what happens to these exploratory therapeutic nanomedicines in vivo following administration, particularly when they are employed in humans. It is important to include in vivo imaging-based tools early in the research phase to aid in the investigation of the potential outcomes following application to individuals, in order to address the information gap and effectively develop therapeutics based on extracellular vesicles (ECV). This will speed up both their development and the development of human trials, enabling the premature distinction of the candidates who are the most eligible to move headfirst. Non-invasive radiolabelled imaging of ECV therapeutics would be a perfect development tool to track and measure ECV biodistribution over time, making it possible to clarify ECVs’ pharmacokinetic characteristics.

## 4. Radiolabelling of ECVs

**Figure 1:**
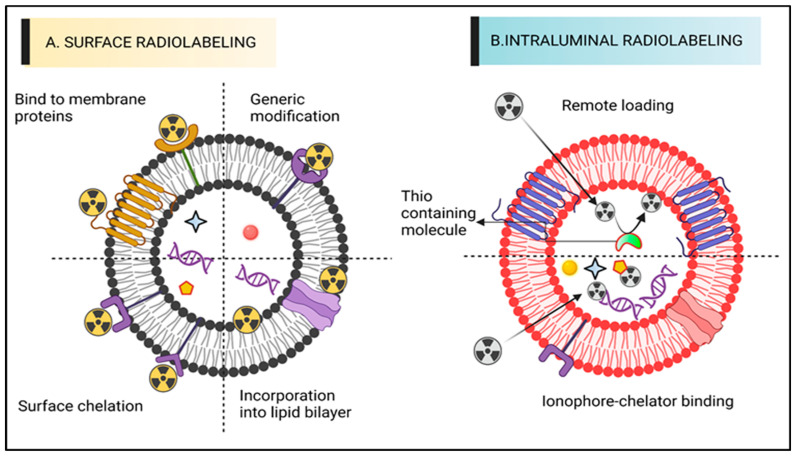
A diagram representing various ECV radiolabelling techniques. (**A**) Surface radiolabelling: a chelator or a radionuclide can be directly introduced into the ECV membrane. (**B**) Intraluminal radiolabelling: radionuclides can be trapped as their lipophilicity changes or bind to biomolecules that chelate metals thanks to ionophores, which allow radionuclides to pass the lipid barrier.

For SPECT and PET applications, ECVs have been labelled with a variety of techniques using radionuclides that release gamma rays or positrons. Figure 1 illustrates types of radiolabelling approaches, and each methodology has been discussed with an emphasis on the benefits and drawbacks that should be recognized when selecting the approach of radiolabelling. This review focuses on the numerous techniques that have been investigated so far for radiolabelling ECVs to enable in vivo tailing with PET, including SPECT envisioning. We evaluate key benefits, incorporating drawbacks of individual techniques, and, when appropriate, discuss them in relation to their in vivo imaging capabilities. We recommend that the reader studies the good articles available [35,36] concerning investigations on imaging ECVs using distinct imaging methods. Due to the similarities in their physical forms, ECVs can be marked by utilizing the identical diverse chemical notions that allow radiolabelling of liposomes [37]. Particularly, both ECVs and liposomes have a comparable size and are made up of an aqueous core that can accommodate a chemical payload and a phospholipid bilayer. In a recent investigation by Man et al., (2019), the many tactics deployed to date to radiolabel liposomal impression devices are described alongside their benefits and drawbacks [38]. We will briefly discuss the two main ways of radiolabelling techniques of ECVs: intraluminal radiolabelling and surface radiolabelling (Figure 1).

Table 1 depicts the overview of the in vivo tracking studies of ECVs and their applications.

### 4.1. Surface Radiolabelling

The most popular technique for radiolabelling ECVs is surface or membrane radiolabelling. This enables the radionuclide to be matched to the proteins of the membrane directly or indirectly via the formation of covalent chemical bonds. To accomplish this, the four tactics displayed below were applied. The four mechanisms of radioactive attachment are genetic adjustment, direct consolidation of radionuclides out of membrane proteins, direct integration into membrane proteins, or radionuclide adhesion via chelator (i.e., radiometals infringing chemical cohort) on the surfaces (Figure 1A). Radionuclide assimilation, which involves the interaction between radionuclides and the elements of the membranes of extracellular vesicles (ECVs) without any specific targeting. In conventional bio-conjugate chemistry, the radiotracer is typically attached to the membrane using surface amine groups through surface chelation methods. The sections following, “Radiolabelling of ECVs using SPECT radionuclides” and “Radiolabelling of ECVs using SPECT radionuclides,” provide more thorough explanations of these techniques along with the relevant radiotracers (vide infra). The ECVs’ surface integrity may be compromised by surface radiolabelling procedures in general, especially if those approaches include chemically altering membrane proteins or significantly changing the makeup of the membrane. Numerous research studies [47] have shown the significance of the proteins and lipids on the surface of ECVs in determining how they behave, and a recent study [48] outlined the numerous techniques for changing the surface of ECVs. The physicochemical characteristics or biodistribution of those meant to permit radiolabelling could thus be significantly modified. These modifications are often implemented to better target tissues or understand ECV function.

Surface radiolabelling of exosomes, which involves the labelling of exosomes with radioactive markers on their surface, has several advantages including, but not limited to:

(i) High sensitivity: surface radiolabelling can achieve high sensitivity, allowing for the detection of low concentrations of exosomes.
(ii) Specificity: surface radiolabelling can provide high specificity by labelling only the surface of exosomes, thus avoiding interference from intracellular components.
(iii) Non-invasive: surface radiolabelling is non-invasive and does not require the use of antibodies, making it an attractive labelling method for clinical applications.
(iv) Easy to perform: surface radiolabelling is a simple and straightforward technique that can be performed with minimal sample preparation.
(v) Versatility: surface radiolabelling can be used in a variety of downstream applications, including imaging, quantification, and tracking of exosomes in vitro and in vivo.

Surface radiolabelling of exosomes involves the use of radioactive labels to tag exosome proteins on their surface. While this technique has been widely used in exosome research, it has some limitations that should be considered. These include:

(i) Radioactive waste: the use of radioactive isotopes for labelling exosomes can generate radioactive waste that must be carefully managed and disposed.
(ii) Limited specificity: surface radiolabelling of exosomes is based on the labelling of surface proteins, which may not necessarily represent the entire exosome population. This technique may not be specific to a particular protein of interest, as it could also label other proteins that are not relevant to the study.
(iii) Potential alteration of exosome behaviour: the labelling process can potentially alter the behaviour and properties of the exosomes, leading to inaccurate results in downstream assays.
(iv) Inability to study internal components: surface radiolabelling only labels the external components of exosomes and, therefore, cannot provide information about the internal contents of the vesicles.
(v) Inability to track exosome movement: surface radiolabelling is a static labelling technique and does not allow for the tracking of exosome movement or dynamic behaviour.

In conclusion, surface radiolabelling of exosomes has some limitations that should be considered when interpreting results. Alternative labelling and tracking techniques such as fluorescent labelling, mass spectrometry, and electron microscopy can provide complementary information to surface radiolabelling [49].

### 4.2. Intraluminal Radiolabelling

Entrapping the radiotracer inside the intravascular space is another way to radiolabel ECVs (Figure 1B). Based on the limited information provided, it appears that the lipid bilayer barrier may act as a protective layer, preventing radionuclides from being trans-chelated by extracellular elements, such as serum proteins. As the radionuclide is exposed during surface radiolabelling, extra-ECV trans-chelation is more likely to take place. For intraluminal radiolabelling of ECVs, the radionuclide must traverse the lipid bilayer or reside inside the ECV. Ionophore-chelator binding and remote loading have both been investigated as ways to do this. The first technique makes use of endogenous intravesicular glutathione, which has the ability to change some complexes from lipophilic to hydrophilic, including Hexamethylpropyleneamine [^99m^Tc]-Tc-hexaoxime ([^99m^Tc]-Tc-HMPAO) [50]. The lipophilic radiotracer complex is transformed into its hydrophilic form after crossing the lipid bilayer membrane and is thereafter confined in the aqueous core of most ECVs. The ionophore-chelator binding approach makes use of popular ionophore ligands, such as tropolone or 8-hydroxyquinolin (oxine), which mix with the radiometals to form a metastable, and thus neutral, complex or allow their transportation across the lipid membrane. Radiolabelling of either cells or liposomal nanomedicines is possible, thanks to the radiometals ability to entangle with metal chelating aggregates included in liposomal cargo [51].

In the ECVs, the radiometal is seen to entangle intravesicular proteins, including nucleic acids. An inability to precisely pinpoint the intraluminal space of the ECVs that the radionuclide entangles will be the underlying flaw of intraluminal radiolabelling techniques, notably those centered on ionophore. Interpreting in vivo imaging can be challenging due to the fact that certain radionuclides, such as radiometals like 64Cu, tend to accumulate in organs that also contain extracellular vesicles (ECVs), such as the liver or spleen. This is particularly true at later time points when ECVs may experience significant lipid bilayer breakage [38]. Both SPECT and PET radionuclides have been used along with the two primary radiolabelling categories, surface and intraluminal, with varying degrees of radiolabelling capability.

This method has several advantages including, but not limited to:

(i) High sensitivity: radiolabelling provides high sensitivity and enables the detection of even small amounts of exosomes.
(ii) Long-lasting signal: the signal from radiolabelling is stable over a long period, allowing for longitudinal tracking of exosomes.
(iii) Non-invasive: radiolabelling is a non-invasive technique that does not require the destruction of exosomes, enabling repeated measurements of the same sample.
(iv) Precise quantification: radiolabelling enables precise quantification of exosomes and their distribution, facilitating the understanding of their biological functions.
(v) Versatility: radiolabelling can be combined with other techniques, such as fluorescence labelling or magnetic resonance imaging, to provide complementary information on exosomes.
(vi) Safety: radiolabelling with short half-life isotopes is considered safe, and the radioactivity levels are usually low enough to avoid any adverse effects.

Some of the disadvantages of this method include, but are not limited to:

(i) Alteration of exosome behaviour: intraluminal radiolabelling can modify the physicochemical properties of exosomes, leading to altered behaviour in vivo. This may cause changes in the distribution, clearance, and cellular uptake of the exosomes.
(ii) Safety concerns: the use of radioactive labels in vivo raises safety concerns, as exposure to radiation can damage biological tissue and increase the risk of cancer. Moreover, the radioactive materials used in radiolabelling can be hazardous and require special handling and disposal procedures.
(iii) Limitations in tracking: intraluminal radiolabelling is limited to tracking exosomes in vivo and does not provide information on the biodistribution or behaviour of the exosomes after they are taken up by recipient cells. This may limit our understanding of the mechanism of action of exosomes and their potential therapeutic applications.
(iv) Technical challenges: intraluminal radiolabelling requires specialized equipment and expertise and may be technically challenging. The labelling efficiency can be low, and the sensitivity of the detection methods used to track the exosomes may be limited.

In all of these, intraluminal radiolabelling of exosomes is a powerful tool for studying the biology of exosomes and their potential clinical applications [49].

**Figure 2:**
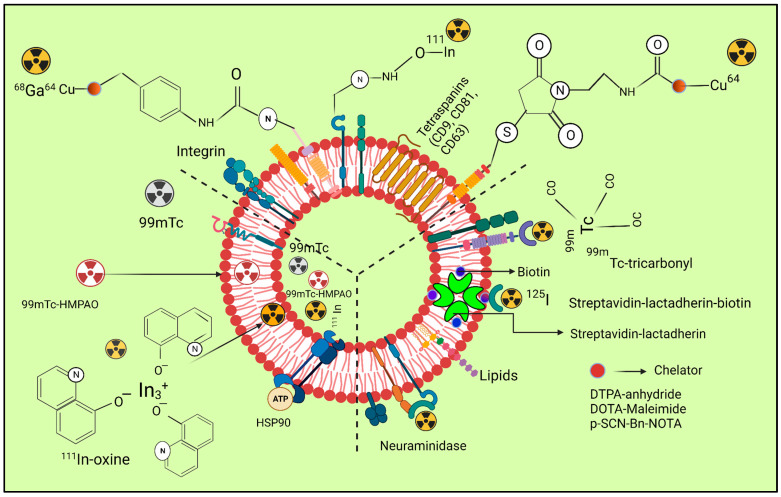
The three radiolabelling examples for extracellular vesicles are shown schematically (ECVs). Bifunctional chelators with a functional group for covalent attachment to reactive amine (-NH2) or thiol (-SH) groups on the surface of ECVs and a metal-binding moiety for radiometal sequestration, such as 64Cu, 68Ga, and o, can be used for membrane radiolabelling. Examples include diethylene triamine pentaacetic acid (DTPA)-anhydride, 1,4,7,10-tetraazacyclo. By intraluminal labelling with lipophilic 111In-oxine or ([^99m^Tc]-Tc-HMPAO) complexes that breach the ECV membrane spontaneously and enter the vesicle lumen, radionuclides can be sealed off. Another method for getting 111In3+ into the exosomal lumen involves the tiny hydrophobic chemical tropolone. Covalent bonds between naturally reactive functional groups or previously added non-native binding groups in ECVs with imaging probes are the basis for covalent binding. Tyrosine residues in neuraminidase-treated ECVs, natural amino acids on the surface of ECVs, and the streptavidin-biotin-lactadherin fusion protein all come together to form stable complexes with radionuclides such as ^124^I and ^99m^Tc. Table 2 depicts the radiolabelling techniques of ECVs by using SPECT and PET radioisotopes.

Figure 2 depicts the radiolabelling of exosomes by using different radio nuclei.

## 5. Radiolabelling of Vesicles That Mimic Exosomes (EMVs)

ECVs’ fluctuation formation, isolation, and radiolabelling qualities can be minimized by utilizing exosome-mimetic vesicles (EMVs). Although they can be made in other ways as well, serial extrusion of cell membranes is the primary method used to make EMVs, and it has been employed for radiolabelling [57]. EMVs’ primary advantage is that they can be designed in larger quantities than ECVs for the mass market. They are identical to ECVs in terms of size, lipid and biomarker expression, protein composition, and tissue aiming prowess. Utilizing mouse macrophage-generated EMVs, Hwang et al. were able to attain an RLY of >93% using [99mTc] Tc-HMPAO (t1/2 = 6 h) (218.8 nm) [25]. The endogenous intra-vesicular thiol factions were employed in lieu of the superficial thiol groups that Banerjee et al. had previously used to transform lipophilic [99mTc]Tc-HMPAO intricate into a hydrophilic form, corralling the radionuclide in the intraluminal site. Mouse macrophage-derived SECVs’ and two distinct types of 99mTc-labelled EMVs’ in vivo biodistribution were studied, and although direct comparisons cannot be made from the photos provided, there seems to have been a emission of the [99mTc]-HMPAO intricate including [99mTcO4]^−^. After, Gangadaran et al., (2018) [55] investigated the biodistribution of EMVs (201.16 nm) produced from RBCs that were 99mTc-labelled. Their theory of radiolabelling is based on the radiolabelling process of RBC, in which intracellular hemoglobin binds to 99mTc4+ reduced by SnCl_2_. In this instance, inside the EMVs, 99mTc is bonded to hemoglobin. Due to the reliability of radiolabelling durability, the team subsequently employed 99mTc-labelled EMVs to consistently radiolabel or analyze white blood cells in vivo [58].

## 6. Innovations in Imaging Techniques for Exosome Tracking In Vivo

Exosomes serve a wide range of nanocarriers for biological purposes for both short- or long-range intercellular communication. They enable the transfer of intricate information among cells, modulating a number of processes such as homeostasis, immunological response, and angiogenesis in both healthy and unhealthy settings. Their extraordinary abilities for motility and targeting, including selective internalization into particular cells, make them desirable delivery vectors. As a result, they offer a possible new area of diagnosis and therapy and could replace cell-based therapeutic strategies. However, this has become a significant obstacle to bringing exosome treatment into the clinic because it is not well understood. More research is needed to fully understand the function of endogenous vesicles within living organisms, as there is currently a lack of sufficient information available. Endogenous vesicles are tiny structures that are enclosed by a membrane. Exosomes can be monitored in vivo to learn more about their biodistribution, migratory potential, toxicity, biological function, communication potential, and mode of action. Therefore the development of exosome tagging and imaging methods that are effective, sensitive, and biocompatible is urgently necessary [59].

**Figure 3:**
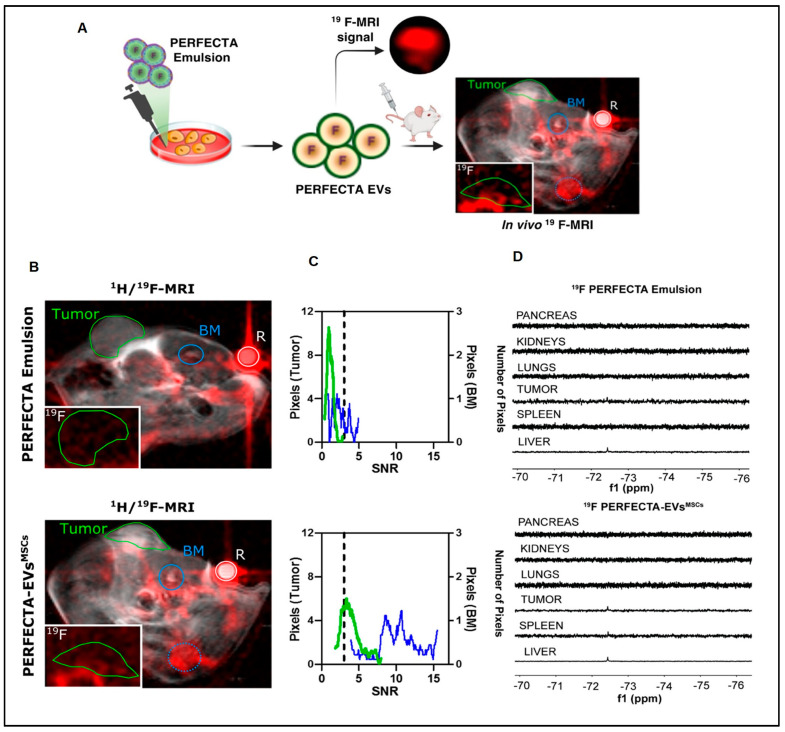
Tumor homing of PERFECTA-ECVs-MSCs. (**A**) In vivo 19F-MRI of BALB-nu mice bearing HeLa tumor: (**B**) merged 1 H and 19F-MRI (red) images from mice after the introduction of PERFECTA emulsion (top) and PERFECTA-ECVs-MSCs (bottom). The green circle indicates the tumor site and the blue circles signify the bone marrow (BM) of the spinal cord (solid) and the knee (dotted). The white circle indicates the standard reference (R). In the white box (bottom-left), 19F-MR image magnification targeted on the tumor mass. (**C**) Histograms of 19F signal-to-noise ratio (SNR) distribution in both tumor and bone marrow of the spinal cord are reported for each mouse. The dashed line identifies the SNR limit above which 19F signal is detectable (SNR > 3). (**D**) 19F-NMR spectra of harvested organs from a representative mouse among those treated with PERFECTA emulsion and those treated with PERFECTA-ECVsMSCs [60] attribution 4.0 International (CC BY 4.0).

Figure 3 describes the in vivo tracking of super fluorinated extracellular vesicles.

## 7. Nuclear Imaging for Exosome Tracking

Cellular envisioning is frequently done via nuclear imaging, which uses radioactive materials to diagnose and cure disorders. Radiation that is emitted by radionuclides can be seen in real time with a specialized camera. Despite the fact that radionuclides have very short half-lives or require comparatively brief times of imaging or tracking, nuclear imaging is able to view deep structures of organs because of its higher sensitivity with tissue penetrating capacity [61]. By incubating the vilifying agents explicitly into exosomes, this approach explicitly infuses the labelling agents into the exosomes. Smyth et al., (2015) [54] state that 3D images are typically acquired using techniques such as single photon emission CT (SPECT) and positron emission tomography (PET). These imaging methods can be combined with anatomical imaging, including MRI, to improve the ability to pinpoint the location of exosomes. Exosomes were labeled with Indium-oxine (111In-oxine) and were infused intravenously. After 24 h, an ex vivo biodistribution examination was performed to observe the accumulation of the labeled exosomes in the body. This method of observation has been previously reported in studies that used fluorescence to compare the accumulation of doxorubicin-loaded exosomes with liposomes in tumors. Our radiolabelling exhibited the speedy circulatory elimination of PC3 exosomes, including MCF-7 exosomes, in PC3 tumor-bearing nude mice. For both exosome types, 5% or less of the initial dose was still present three hours post-injection. It’s interesting to note that tumor-bearing mice and healthy mice both experienced rapid exosome clearance. To track labeled exosome-mimetic vesicles produced by red blood cells, they were labeled with 99mTc. Gangadaran et al., (2018) [55] adopted a varied imaging technique. At 1 and 3 h after intravenous injection, they used a gamma camera to do in vivo imaging on mice with tagged exosomes. The liver and spleen had higher uptake than the thyroid, which had no uptake, whereas the thyroid, stomach, and bladder had the highest concentrations of free 99mTc signal. Fluorescence and immuno-FLI were used to confirm their radiolabelling findings [55]. Faruqu et al., (2019) [43] utilized both intraluminal labeling and membrane labeling techniques to label a diverse isotope (111Indium) in their study, where they employed a bifunctional chelator that was covalently linked for the chelation of 111Indium. Exosomes from melanoma cells that were radiolabelled (B16F10) were employed to assess the biodistribution of the particles in mice with healthy vs. damaged immune systems. When membrane-labelled B16F10 exosomes were intravenously delivered, imaging of the whole-body showed that the exosomes had a significant lung buildup at one hour after delivery, which subsequently gradually decreased over the following 4 to 24 h.

## 8. Use of Radiolabelled Exosome for Imaging and Quantitative Biodistribution

Hwang et al., (2015) [25] developed a technique to label macrophage-derived exosome-mimetic nanovesicles (ENVs) with 99mTc-HMPAO under physiological conditions, in order to track the distribution of 99mTc-HMPAO-ENVs in live mice. The murine RAW264.7 macrophage cell line was used to create ENVs, which were then labelled with 99mTc-HMPAO for 1-h incubation before the free 99mTc-HMPAO was removed. “After being labelled with 99mTc-HMPAO, 99mTc-HMPAO-ENVs had radiochemical purity over 90%, while the utterance of the exosome explicit protein (CD63) was unaffected”.

After being labeled with 99mTc-HMPAO, the 99mTc-HMPAO-ENVs demonstrated a radiochemical purity of over 90%. The expression of the exosome-specific protein CD63 was not impacted. The serum stability of 99mTc-HMPAOENVs was high (90%) and comparable to that of phosphate-buffered saline for the first five hours. In contrast to mice treated via 99mTc-HMPAO, which exhibited significant brain retention till 5 h after injection, animals treated with 99mTc-HMPAO-ENVs showed enhanced liver uptake with negligible uptake in the brain on SPECT/CT imaging. In order to enhance exosome fabrication, melanoma cells (B16F10) were cultivated in bioreactor vesicles. Faruqu et al., (2019) [43] investigated the exosomes’ radiolabelling for a comparison of biodistribution exploration in immune-competent vs. immune-deficient mice. After being ultracentrifuged onto a single sucrose cushion, the exosomes (ExoB16) derived via B16F10 were isolated then examined. ExoB16 was radiolabelled utilizing membrane labelling and intraluminal labelling, which involved trapping _111_Indium through tropolone shuttling (_111_Indium chelation via DTPA-anhydride, a bifunctional chelator with covalent attachment). Utilizing gel filtration with thin-layer chromatography, labelling effectiveness and stability were evaluated.

Radiolabelled ExoB16 (1 × 1011 particles/mouse) was administered intravenously to melanoma incurring, C57BL/6 immuno-competent and immune-deficient NSG mice. The radiolabelling reliability or radiochemical resilience of membrane-labelled ExoB16 was higher than that of intraluminal denoted exosomes (4.73 ± 0.39% with 14.21 ± 2.76%, respectively), showing greater radiolabelling stability (80.4 ± 1.6%) or radiolabelling efficiency (19.2 ± 4.53%). Utilizing the membrane vilifying perspective, ExoB16 in vivo biodistribution in melanoma incurring C57Bl/6 mice was explored. ExoB16 was found to accumulate the most in the liver and spleen, accounting for 56% of the total accumulation with 38% ID/gT. The kidneys showed a lower accumulation of 3% ID/gT. Exosome membrane radiolabelling is a dependable method that enables quantitative biodistribution investigations and accurate live imaging on possibly all exosome kinds without modifying parent cells. The utilization of [_89_Zr] Zr (oxinate)-4 (a corpus or liposome radiotracer) for prompt or intra-luminal radiolabelling of distinct ECVs was examined by Khan et al., (2022) [56] in order to achieve elevated radiolabelling yields. It appears that a study was performed to refine radio synthesis and radiolabelling methodologies for ECVs (extracellular vesicles) in order to prevent their deterioration and optimize their characteristics. The study utilized various in vitro techniques such as dot blot, cryoEM, nanoparticle monitoring evaluation, and flow cytometry, and employed PANC1 (pancreatic cancer) ECVs in a physically fit experimental mouse model. The study demonstrated that it was possible to follow the ECVs that were labelled with 89Zr in vivo using PET imaging for up to 24 h and consistently track them within this time frame. Additionally, it was noted that purposely heat-damaged ECVs had much lower spleen absorption as compared to undamaged ECVs in terms of biodistribution. This radiochemical technique will aid research into the in vivo behavior of ECVs and provide answers to fundamental biological problems, supporting as delivery systems, biomarkers of disease (such as identifiers of metastatic habitats), or therapies [53].

In this investigation, various types of cells were studied, including tumor cells that have or have not undergone treatment, myeloid-inferred suppressor cells, and progenitor cells of endothelial cells. The objective was to understand how to measure and visualize the in vivo distribution of exosomes marked with radioisotope 131I in these different cell types. The exosome-based targeted therapy and disease progression can be tracked using the implemented in vivo envisioning approach. Exosomes can be radiolabelled to examine their biodistribution in vivo; Faruqu et al., (2019) [43] set out to create an innovative, trustworthy, and all-encompassing technique for this. With the use of electron microscopy, flow cytometry, nanoparticle tracking evaluation (NTA), and protein assays, exosomes (ExoB16) synthesized from B16F10 were isolated by ultracentrifugation onto a single cushion of sucrose. ExoB16 was radiolabelled via membrane labelling and intraluminal labelling (_111_Indium was trapped through tropolone shuttling) (chelation of _111_Indium utilizing DTPA-anhydride, a bifunctional chelator exhibiting covalent attachment), utilizing gel filtration and thin-layer chromatography, and labelling effectiveness and stability were evaluated. At 1, 4, and 24 h after intravenous injection of radiolabelled ExoB16 (1 × 1011 particles/mouse) into melanoma incurring immunocompetent (C57BL/6) and immunodeficient (NSG) mice, metabolic cage findings, complete body SPECT-CT envisioning, and ex vivo gamma tallying were carried out. The intraluminal marked exosomes (4.73 ± 0.39% including 14.21 and 2.76%, respectively), in contrast to the membrane-marked ExoB16, evidenced enhanced radiolabelling potency and radiochemical resilience (19.2 and 4.53% including 80.4 and 1.6%, respectively). Exosome membrane radiolabelling is a dependable method that enables quantitative biodistribution investigations and accurate live imaging on possibly all exosome kinds without modifying parent cells. For tagging and tracking ECVs, various fluorescent imaging techniques have different advantages and disadvantages (Table 3) [62].

## 9. Radiolabelling Including In Vivo SPECT/PET Imaging Problems with ECVs

The ECVs’ volatility is one of the main obstacles to in vivo imaging. According to a study by Clayton et al., (2003) [63], ECVs have a high concentration of CD55 and CD59, which may help them survive longer in-vivo. The ECV radiolabelling experiments, as well as those using non-radiolabelled ECVs, showed that the half-life of intravenously injected ECVs was very short, as brief as two minutes [64]. Even after ECVs have been eliminated from the circulation, they are still persistent, primarily in the liver or spleen and in the reticuloendothelial system (RES) organs. The bladder accumulates the most imaging signals after the liver and spleen, which is only conceivable if the ECVs can get through the kidneys’ glomerular filtration. Choi et al., (2007) [65] used quantum dots to show that kidneys often do not pass nanoparticles larger than 8 nm. These findings, including information on antibody clearance, imply that the kidney filtration threshold size may be akin to minute proteins. Clearance of renal preserved antibodies is presumed to be unimportant since the size of a reminiscent antibody is significantly larger than the threshold of glomerular filtration (55 kDa) [66]. Contrarily, antibody fragments are significantly smaller and eliminated by the kidneys (the IgG Fab fragment is about 50 kDa in size, for instance). Therefore, it appears to us that the rapid ECV disintegration in blood/serum is likely to be the cause of the renal/bladder intake exhibited in numerous radiolabelled ECV SPECT/PET envisioning research findings. According to earlier studies, ultracentrifugation can cause serum proteins (such as albumin) to co-precipitate with less pure serum proteins, as evidenced via the particle-to-protein ratio. The utilization of instantaneous thin-layer chromatography (iTLC) to discern radiochemical resilience in blood is a key problem with many of the ECV radiolabelling experiments cited above.

## 10. Thiol-Michael Inclusion Loads Imaging Probes Including Drugs onto Exosomes

The dimensions and integrity of exosomes (EXOs) were unaffected by additional chemical engineering, with the exception of chemical transfection and the previously stated saponin-incorporated drug freight on the exosomes (EXOs). An a, b-unsaturated carbonyl reacts with an enolate-type nucleophile in the Michael addition. It has been used for a long time in organic synthesis to create highly selective compounds in a safe manner [67]. Chemical reactions of this nature have evolved into highly efficient and adaptable procedures, and it appears to have emerged roughly concurrently with “click chemistry” in materials science. To produce a highly effective reaction, the thiol-Micheal addition is intended to proceed in conditions that are mild and solvent-free. Sulfhydryl groups (-SH) are widely distributed in the majority of membrane proteins found on exosomes (EXOs). These groups can be utilized as a binding link for drug loading through the use of maleimide-containing sulfhydryl strings. Numerous efforts have been made to date to put thiol-Michael addition on the exosomes (EXOs) in practice. For instance, Roberts-Dalton et al., (2017) [68] used the maleimide/thiol progression to bind Mal-Alexa 633 and MalAlexa488 on the EXOs produced by prostate malignant cells in order to describe the endocytosis of EXOs by living cells. This chemical method undoubtedly gave the exosomes (EXOs) more versatility when it came to fluorescent labelling. The fluorescent signals allowed them to rapidly observe their endocytic traffic and interactions with cells [68]. By using thiol-Michael addition, quantum dots (QDs) were affixed to the exosomes (EXOs), in addition to fluorophores, also enabling a gentle and biocompatible labelling method. This study used maleimide or biotin to functionalize a DNA hinge at each terminal. The chemical interaction between the DNA’s built-in maleimides and the thiols on the exosomes (EXOs) subsequently enabled the DNA fragment to be coupled on the M1 macrophage-originated exosomes (EXOs). The terminal biotin of the DNA hinge was then easily realized by the streptavidin-labelled QDs to combine to EXOs for tumor scanning. Another component of thiol-maleimide interaction focuses on its use in disease therapies in addition to in vivo imaging [69]. Due to the availability of maleimide-thiol interactions, the addition of a novel group of attraction ligands on exosomes (EXOs) in this manner is getting a lot of consideration in focused therapy. Han et al. used thiol-Michael addition to bind aptamers to the HEK293T cell-derived exosomes (EXOs) in order to identify prostatic cancer cells [70].

### 10.1. Loaded on Exosomes Include Imaging Probes and Medications via Different Chemical Engineering

Numerous studies have shown that EXOs are being modified via chemical engineering to function as “bioscaffolds” for medical diagnosis and treatment. For the early-stage diagnosis of malignancy, the detection of intact EXOs is essential because they behave as desirable substitutes for certain tumor cell-associated counterparts [71]. In a recent ground-breaking study of the introduction of fluorescent reporters and the development of EXO-binding cavities, molecular imprinting was developed. In one investigation, nitrilotriacetic acids (NTAs) and the NiII complex were used to initially bind the His-adhered G protein to the Au (gold) substrate. Then, anti-CD9 antibodies were immobilized to bind to protein G that had been His-tagged in order to get CD9 elevating EXOs expressed. The surface of EXOs was altered via a bridge of disulfide utilizing thiolated oleyl poly (ethylene glycol), dithio-ethyl-methacrylate including 2-(2-pyridyl) after they had been distinctly captured through antigen/antibody binding. After that, a polymer matrix imprinted the collected EXOs. When the pattern EXOs are removed, the bridge of disulfide transforms into an available thiol in the binding pockets of EXO, making sure that the fluorescent labels are site-discreet conjoined to the thiol class. By observing the fluorescent signal in the EXO-binding pockets, it was possible to determine that CD9-specific and undamaged EXOs had been successfully detected [72].

### 10.2. Assessing the Biodistribution Including Exosomes PK Utilizing Bioluminescence with Fluorescence Imaging Approaches

Exosomes are typically labelled with different lipophilic fluorescent dyes or luminous probes in order to assess their in vivo properties. In Wiklander et al., (2015) [73] and Peinado et al., (2012) [74], exosomes were derived from B16F10 murine melanoma cells and labeled with fluorescent dyes, specifically DiR and PKH67. In a study conducted by Grange et al., (2014) [75], the localization of exosomes in various organs including bone marrow, liver, lung, and gastrointestinal system was examined using exosomes that were labeled with a near-infrared (infrared) dye known as DiD. The results of the study showed that the amount of exosomes derived from mesenchymal stem cells (MSCs) that were deposited in the renal system was significantly higher in animals that had been administered with acute kidney injury (AKI) compared to healthy mice [75]. By employing the direct incubation method to tag exosomes from prostate cancer cells with both DiD and OG-Paclitaxel (PTX), Saari et al. were able to show cancer targeting effects [76]. To assess exosome distribution at the cellular level, tetraspanins and other exosomal surface proteins were labelled. Exosomes generated by breast cancer cells during the metastatic process must be identified, as well as those that aim the stroma at metastatic sites. Suetsugu et al., (2013) [77] employed CD63 that was green fluorescent protein (GFP)-tagged. Since luminescence does not rely on external light sources to emit light, it has a greater signal-to-noise ratio than fluorescence labelling. To analyze the distribution of exosomes across different biological tissues, the researchers utilized a fusion protein called gLuc that incorporates LA. The C1C2 domain of LA has the capability to merge with exosome membranes, and this property was leveraged in the study. By using gLuc, the researchers were able to examine the presence of exosomes in various tissues [78]. According to Lai et al., (2014), it was also utilized as a gLuc platelet-derived growth-factor receptor (PDGFR) transmembrane region fusion protein to track exosomes during in vivo MRI [33]. Additionally, exosomes could be marked with contrast agents (such as superparamagnetic (SP) iron oxide nanoparticles (IONs), or USPIO) using techniques including electroporation and co-incubation [79,80]. The ferritin heavy chain (FTH1) fusion protein, which is an MRI contrast agent and lactadherin, could also be utilized to mark exosomes for MRI imaging [81]. Although MRI is less sensitive than radioisotope-based imaging, it offers the advantages of being radiation-free and having spatial resolution for deep tissues in addition to superior soft tissue contrast.

## 11. Luminescence-Based Aptasensor

The process through which a substance emits light without requiring or producing heat is known as luminescence. In order to monitor exosomes, numerous aptasensors based on luminescence have been created because of their great sensitivity, because they are relatively inexpensive, and because they are simple to control. Electrochemiluminescence and luminescence resonance energy transfer (LRET) are the two kinds of luminescence that are distinguished here based on the energy source (ECL).

### 11.1. Transfer of Luminescence Resonance Energy

Aptasensors depending on LRET display the same positive effects of high sensitivity, excellent operability, and resolution as the FRET mechanism. Therefore, utilizing rare earth incapacitated up-conversion nanoparticles (UCNPs) that are transformed into gold (Au) nanorods is essential. Chen and colleagues (2018) [82] created a simple and user-friendly aptasensor based on paper-supported LRET using gold nanoparticles (NRs). The sensor employed two aptamer sections of CD63, one for capturing and the other for detecting. The aptamers allowed exosomes to recognize and conjugate with each other, leading to the bridging of paper upconversion nanoparticles (UCNPs) via the capture probe and Au NRs via the detection probe. This bridging induced LRET, resulting in quenching of green fluorescence.

### 11.2. Electrochemiluminescence

The ECL approach, in contrast to electrochemical exploration, does not rely on external parameters, including temperature, electrode surface roughness, pH, humidity, and many more. Qiao et al., (2019) [83] created an aptasensor for electrochemiluminescence (ECL) by functionalizing an aptamer onto a glassy carbon electrode. The electrode was then covered with ECL emitters comprising of CdS nanocrystals incapacitated with Eu+++ and modified with mercaptopropionic acid. This approach was employed to avoid interference from background currents [83]. A potent ECL signal including 37 particles/μL, a minimal LOD, was produced by the ECL aptasensor built by Fang et al., (2020), utilizing Ru (dcbpy)3/2+ as co-reactors and a pack of antibody/MXenes and aptamer\GCE exosome utilitarian zed with quantum dots of black phosphorus working as a signal amplifier [84]. The possibility of using this sensor to detect exosomes via a thermometer is notable.

### 11.3. Strip Aptasensor

On paper-based portable devices, the aptasensor, lateral flow-strip (LFS) is frequently used and has a simple user interface and color and signal change makes the outcome clear. LFS is frequently separated into 2 categories of pack strip, with either competitive strip grounded on various exchanges onto test lines [Cheng et al., 2019b]. Based on thermal signals, Cheng et al. created a sandwich strip for exosome identification. Yu et al., (2020) [85] suggested evidence-of-concept for the exosome detection of a competitive strip to expedite the experimental procedure [85]. The test mark provided the data directly, without the control line, which was done on purpose. When exosomes were absent, the test line turned red because the CD63 aptamer connected to Nano gold and hybridized with its corresponding strand there. As a result, the relationship between exosome quantity and color was inverse. The LFS showed traits including simple batch production, long-term stability, a need for few samples, affordability, and effectiveness; it was further appropriate for experimental application.

### 11.4. Surface Enhanced Raman Scattering Aptasensor

SERS has a constrained spectral bandwidth and distinctive fingerprint properties for distinguishing different exosomes. MB-capture substrates by utilizing diverse aptamer probes. Wang et al. advanced a SERS aptasensor for simultaneous finding of multiple exosomes [86]. To collect common exosomes, the CD63 aptamer was embellished with gold shell magnetic nanobead substrates. Targeting exosomes with specific aptamers modified AuNPs and a Raman correspondent as a readout signal foundation, establishing a pack of MB-exosome AuNPs, which were the foundations for the SERS identification.

### 11.5. Surface Plasmon Resonance Aptasensor

SPR is an approach to ocular biosensing that can be utilized to observe in-situ and real-time biomolecular interactions on metal surfaces. This technique doesn’t require any markers and doesn’t harm biomolecules in any way. For the purpose of exosome detection and dual AuNP-assisted signal amplification, Wang et al. used the SPR aptasensor for exosome uncovering, then dual AuNP-aided signal amplification [87]. In order to catch generic exosomes, CD63 aptamers were briefly immobilized on Au film surfaces.

### 11.6. Giant Magnetoresistance Aptasensor

GMR sensors are gaining significant attention in the field of biosensors due to their compatibility with IC technology, including their ability to work seamlessly with superparamagnetic nanoparticles. This compatibility makes GMR sensors an attractive option for biosensor applications. Zhu et al., (2019) [88] established a GMR aptasensor for exosome finding grounded on the magnetic signal reply of MoS_2_-Fe_3_O_4_ nanostructures (MOFE). On the GMR sensor surface, the CD63 aptamer was modified for widespread exosome capture. To recognize exosomes, MOFE-labelled aptamer probes were used. This pack of “GMR/CD63 aptamer-exosome-MOFE/detection aptamer” greatly increased the signal of GMR (Figure 3).

## 12. Exosome Tracking In Vivo and In Vitro

Through in vivo and in vitro research, real time drug delivery systems must be watched to demonstrate the drug’s structure, concentration, and timing of arrival at the target site. Animal application experiments require the tagging and monitoring of exosomes. The exosomes of sick tissues were observed using computer tomography (CT), fluorescent bioluminescence imaging, microscopy, and magnetic resonance imaging (MRI) [89]. Fluorescence microscopy is a crucial tool for understanding the physiological processes of living cells and, currently, optical envisioning is an arguably popular envisioning approach for studying cells including molecules. To determine the association between the exosomes and the cells examined, fluorescent probes are used to initially label the exosomes. When enticed, the specimens being assessed are examined based on fluorescence signals that are emitted, and the physiological evolution of specific cells may be followed. Exosome fluorescence tagging techniques fell into four groups that were widely used by many people. These include:

(1) Natural dyes: the surface of the exosome lipid bilayer receives the fat chain through the lipophilic organic dye. This characteristic makes it possible to prolong and stabilize the fluorescence of the fluorescent dye. In addition, the fluorescent dye’s emission spectrum, which extends from 502 from 734 nm, has a broad application window with great selectivity. However, the fluorescence quantum yield, half-life, light color, and other properties of this organic dye differ between dyes. Moreover, variations among dyes may involve the mutual reinforcement of other liposomes through dyeing, which will contribute to imprecise results [90].
(2) Fluorescent proteins that are genetically encoded: red and green fluorescent proteina, as well as other fluorescent proteins fall under this category. Through the use of gene editing technologies, they are alluded to as marker proteins found on the superficial exosome. To assess the in vivo and in vitro movement and metabolic transfer of exosomes and prevent other liposomes from being mislabelled, one of the exosome markers, CD63, was frequently fused with them [77].
(3) Reporter molecules that are immunofluorescent: the highly specific immunofluorescence labelling technique involves colouring the exosome first, then the antibody with an organic dye. The need for microscopy equipment is quite high because exosomes are so tiny. Due to their increased resolution, stochastic optical reconstruction microscopy and photoactivated localization microscopy were better suited for observing exosomes [91].
(4) Nanomaterials that glow: due to their tiny size and superior optical qualities, fluorescent nanoparticles are uncomplicatedly bound and don’t affect the physiological activity of exosomes in any significant way. However, there is a great mandate for near infrared (IR) fluorescent nanoprobes including deep tissue dissemination and negligible autofluorescence background tracking [92]. The primary application of bioluminescence imaging is monitoring biological activity. Its key benefits include great sensitivity, the absence of a light source, and the weak background signal. Utilizing only oxygen as the catalyst for the reaction of their specific substrate, luciferases produce bioluminescence. Exosomes can be accurately distributed using this technique in both vitro and in vivo; transient indications cannot be utilized to trace exosomes for an extended period. Numerous studies have utilized the labeling of extracellular vesicles (EVs) with Gaussia luciferase, which is a protein that produces light, allowing for non-invasive bioluminescence imaging procedures to monitor the distribution and characteristics of EVs in vivo, since EVs are very small in size, measured in nanometers [93]. With MRI, it is possible to penetrate deep tissues and obtain superb 3D images. In the picture contrast, different hydrogen nuclei’s relaxation durations in the tissue environment are depicted. In order to elevate chemical contrast, imaging sensitivity mediators such as SPIONs stay widely utilized. Tracking of C57BL/6 experimental mouse models was done through magnetic resonance imaging.

One study electroporated SPIONs into exosomes [94]. Researchers frequently use CT, a high-resolution imaging tool for medical applications, for exosome tracing. The distinctive X-ray absorption in the tissue component is utilized to extract a 3D image made up of distinct cross-sectional images. Similarly, contrast agents are required for the backing of CT visualization signals. Due to the significant X-ray attenuation, gold (Au) nanoparticles (GNPs/Au-NP) are presently a very prevalent divergent agent. An article described how to label GNPs with a glucose coating. GLUT-1 is a protein that facilitates the entry of glucose-coated GNPs into cells derived from MSCs and aids in energy absorption within the body. Its primary function is to transport glucose molecules across the cell membrane. GLUT-1 is expressed in various cells throughout the body and is crucial for maintaining glucose homeostasis. Then, CT was utilized for non-invasive in vivo neuroimaging with tracking of exosomes [95]. The exosome-like nanovesicles were labelled with 99mTc-HMPAO radioactive material under physiological conditions. Following intravenous injection (iv) into BALB/c mice, the 99mTc-HMPAO-ENVs distribution in vivo was monitored via SPECT/CT [28].

In research conducted by Lázaro-Ibáñez, the impact of five different tracers on the biodistribution of extracellular vesicles (ECVs) in vivo was investigated. The aim was to develop ECVs as effective drug delivery vehicles and therapeutic agents while being able to track them without disturbing their biodistribution. The tracers evaluated included a noncovalent fluorescent dye (DiR), covalent modification with _111_indium-DTPA, bioengineering with fluorescent (mCherry) or bioluminescent (luciferase) proteins, and a commercially available iron oxide nanoparticle (Feraheme). The study concluded that none of the labelling methods significantly influenced the biodistribution of ECVs when compared to the control group that was not labelled. However, covalent labelling could potentially affect the functionality of ECVs, and further investigation is required to determine the most suitable labelling technique for different ECVs applications [96] (Table 4).

## 13. Radiopharmaceuticals for Theranostic Applications

Radiolabelled nanoformulations have been the subject of extensive research for their potential applications in theranostics. Metallic, polymeric, liposomal, dendritic, and silica nanoparticles are among the various types of radiolabelled nanoformulations that have been developed and evaluated for their therapeutic and diagnostic capabilities.

### 13.1. Radiolabelled Metallic Nanoparticles

Metallic nanoparticles are very small metal particles that range in size from 1 to 100 nanometers. Due to their size and surface characteristics, they can function effectively as catalysts. The large surface area-to-volume ratio, quantum confinement, increased number of kinks, and plasmon excitation is some of the unique features of metallic nanoparticles that make them useful in various applications. Metallic nanoparticles have several benefits, such as their ability to enhance Rayleigh and Raman scattering, strong plasma adsorption, and imaging of biological systems. However, their commercial use is limited due to their instability, potential impurities, toxicity, and carcinogenicity. Nanoparticles have emerged as promising tools for both imaging and therapy of cancer cells. Researchers have developed various hybrid nanoparticulate systems utilizing gold and iron-based materials. Among the commonly used nanoparticle types are radiolabelled superparamagnetic nanoparticles, quantum dots, and gold nanoparticles. These nanoparticles can be surface-modified with specific aptamers, antibodies, or proteins to make them target-specific, thereby facilitating inspection of cellular changes or monitoring interactions by changing signal-to-noise ratios using imaging techniques such as PET, SPECT, or hybrid imaging [106,107].

### 13.2. Radiolabelled Gold Nanoparticles for Theranostic Application

Gold nanoparticles possess unique physicochemical properties that make them excellent candidates for use as nanocarriers in a variety of biomedical applications. They have a low reactivity, good binding affinity, high surface area, optimal size, good stability, and biocompatibility. Due to their chemical inertness, biocompatibility, and ease of modification, they can be used for both therapeutic and diagnostic purposes. Furthermore, these nanoparticles are non-toxic and can achieve passive as well as active targeting [108].

The study explored the use of radionuclide-embedded gold nanoparticles for optical imaging of dendritic cell (DC)-based immunotherapy and tracking of DC migration to lymph nodes. Gold nanoparticles modified with oligonucleotides were used for PET/CT imaging, which showed higher radio sensitivity and allowed for long-term monitoring of DC migration to lymph nodes. In vivo experiments on mice immunized with Lewis lung carcinoma demonstrated strong antitumor immunity for lung cancer without any adverse effects on the biological function of dendritic cells. These findings suggest that radionuclide-embedded gold nanoparticles have the potential to be an effective tool for cancer immunotherapy [109].

The study also involves the creation of a hybrid system for the treatment and diagnosis of lung cancer, which utilizes PEGylated gold nanoparticles to target EGFR. Cetuximab is also conjugated to the gold nanoparticles for therapeutic purposes. Radio-immunotherapy is employed using the radioisotope ^131^I, which is attached to the gold nanoparticles using the Iodogen method. The resulting system is designed to selectively target and treat lung cancer cells expressing EGFR, while also allowing for the imaging and monitoring of the tumor. The in vivo study conducted on tumor-bearing mice showed promising results, including anti-proliferative activity and radioactive retention in the tumor, indicating the potential of the system as an effective nano theranostic for EGFR-expressing lung cancer [110].

Researchers studied the potential of a hybrid theranostic nanosystem to precisely locate and diagnose vulnerable atherosclerotic plaques by targeting apoptotic macrophages. They used gold nanoparticles that were radiolabelled with 99mTc and conjugated with Annexin V as targeting molecules, which were synthesized through a chelation method. The nanosystem was evaluated using SPECT/CT imaging, and promising results were obtained, indicating its potential as a tool for identifying and assessing vulnerable atherosclerotic plaques [111].

This study utilized gold nanoclusters that were modified with AMD3100 (plerixafor) and labelled with _64_Cu to image primary tumors and lung metastases in a mouse model of _4_T1 orthotopic breast cancer. The PET imaging results obtained after administering the developed formulation demonstrated accurate detection of CXCR4 up-regulation, which confirmed the nanoclusters’ targeting specificity. Therefore, the study concluded that this approach holds promise as a diagnostic tool for early and accurate diagnosis of breast cancer with lung metastasis [112]. Gallic acid, a natural compound with anti-cancer properties, was combined with gold nanoparticles to develop a drug delivery system for intra-tumoral administration of the anti-cancer drug doxorubicin (DOX). The nanoparticles were also radiolabelled with 99mTc to facilitate imaging. In vitro experiments on breast cancer cells (MCF-7) showed that the gallic acid-gold nanoparticle system enhanced the effect of DOX, resulting in a four-fold reduction in the IC_50_ compared to DOX alone. While these results are promising, it is important to note that further studies are necessary to assess the safety and efficacy of this system in humans, including in vivo studies and clinical trials [113].

### 13.3. Radiolabelled Magnetic Nanoparticles for Theranostic Application

The field of nanotechnology has made significant strides in the synthesis and physicochemical properties of magnetic nanoparticles, which have proven to be attractive candidates for various biomedical applications. Magnetic nanoparticles offer high surface area and improved quantum efficiencies, making them useful as nanocarriers for molecular imaging and therapeutic applications. Moreover, the release of drugs from these nanoparticles can be readily modified using external fields such as magnetic or NIR radiation. Another promising application of nanoparticles is in Surface Enhanced Resonance Raman Scattering (SERRS), which enables accurate detection of malignant or premalignant lesions and non-invasive imaging of the whole body. In light of these advantages, researchers have developed SERRS nanoparticles labelled with _68_Ga using a chelator-free method for imaging pre-operative and intraoperative profiles in lymph node disease using PET imaging. This technique shows promise for improving the accuracy and sensitivity of imaging in lymph node disease, thereby aiding in more effective treatment planning and monitoring.

The study aimed to investigate the passive targeting efficiency of 64Cu-labelled PEGylated and reduced graphene oxide-iron oxide (GO-IO) nanoparticles via the Enhanced Permeability and Retention (EPR) effect, using photoacoustic and PET imaging techniques. The in vivo study was conducted on a hind limb ischemia model, which demonstrated that the hybrid radiolabelled metallic nanoparticles were able to effectively target the ischemic tissue through passive targeting via the EPR effect, as evidenced by clear visualization of the nanoparticles in the affected area [114]. Researchers have developed a theranostic system using superparamagnetic iron oxide nanoparticles (SPIONs) labelled with 223Ra for targeted α-particle therapy (TAT) to investigate its potential for application in tumors. The system displayed good in vitro stability in various biological media, and its stability in bovine serum was deemed acceptable [115]. The study explored the potential use of PEGylated superparamagnetic iron oxide nanoparticles (SPIONs) that were radiolabelled with the isotope 69Ge for hybrid imaging through PET/MRI in sentinel lymph nodes. The study demonstrated the direct and efficient radiolabelling of _69_Ge on the nanoparticles without the use of a chelator. The results showed that PEGylated SPIONs accumulated over time in tumor cells and were found to be safe and biocompatible for use in hybrid imaging and therapy for cancer [116]. The study discusses the creation of a novel dual-modality imaging probe that can be used for non-invasive visualization of angiogenesis. The probe is made up of two components: paramagnetic nanoparticles coated with 2,3-dimercaptosuccinic acid (DMSA-MNPs) and a PET _68_Ga chelator, NOTA derivative, which is coupled with the GEBP11 peptide. Immunofluorescence and Prussian blue stains were used to demonstrate the peptide’s affinity, indicating its potential in visualizing plaque formation [117].

### 13.4. Radiolabelled Silica Nanoparticles for Theranostic Application

Silica nanoparticles are popular for drug delivery and bio-imaging due to their advantages, but they have some drawbacks. In a recent study, researchers developed mesoporous silica nanoparticles with MUC1 aptamer capping, loaded with safranin O, and functionalized with aminopropyl groups for controlled drug delivery and tumor imaging. The system demonstrated higher tumor uptake of nanoparticles in mice, showing a promising approach for simultaneous drug delivery and imaging [118]. The study investigated the use of peanut-shaped silica-coated graphene oxide nanoparticles loaded with gambogic acid and radiolabelled with _188_Re for tumor targeting. The results demonstrated that _188_Re provided radiotherapy and SPECT/CT imaging in a VX2 tumor model. Additionally, the peanut-shaped nanoparticles showed higher tumor uptake than spherical-shaped nanoparticles [119]. The study assessed the potential of 99mTc surface-modified manganese oxide-based mesoporous silica nanoparticles as theranostics for cancer. In vivo administration of the nanoparticles in tumor-bearing mice showed higher T1-MRI relativity with simultaneous delivery of anti-tumor drug DOX at tumor sites. This suggests that this approach has great potential for cancer diagnosis and therapy [120]. A dual-modality probe was developed for targeted imaging of breast cancer cells that overexpress HER-2. The probe consisted of a silica nanoparticle functionalized with polyamidoamine and anti-HER-2 antibodies and radiolabelled with 99mTc. To radiolabel the nanoparticles with the chelating agents DTPA and MAG3, stannous chloride was used. This dual-modality probe has the potential to improve the specificity and sensitivity of breast cancer imaging [121].

### 13.5. Radiolabelled Polymeric Nanoparticles for Theranostic Application

Polymeric nanoparticles have become popular for radiotheranostic applications in diseases such as cancer, diabetes, and neurological disorders. Radiolabelling can be done through pre- or post-labelling strategies using chelators or cross-linking agents. A recent study used radiolabelled HPMA nanoparticles loaded with antitumor drugs to evaluate their efficacy in an in vivo tumor model, showing improved accumulation in tumor cells, longer circulation time, and a higher therapeutic index with the combination of radiotherapy and chemotherapy [122]. The study investigated the use of radiolabelled nanoparticles for tumor angiogenesis therapy. Poly (HPMA) nanoparticles were radiolabelled with _64_Cu using DOTA as a chelating agent and conjugated with target-specific peptide RGDyK for tumor localization using PET imaging. Additionally, _89_Zr-radiolabelled HPMA polymeric conjugates were prepared for therapeutic efficacy on a _4_T1 cancer cell model. The study found that the disparity and molecular weights of the polymer played a significant role in the pharmacokinetic profile of polymeric conjugates. Furthermore, higher molecular weight polymeric carriers showed higher cellular uptake and cytotoxicity in vitro [123]. The study developed HPMA copolymer conjugates functionalized with site-specific peptide RGD4C, labelled with 99mTc and _90_Y isotopes for targeted radiotherapy of neoplastic cells. The nanoconjugates induced higher apoptosis in the targeted cells with no side effects on off-targeted tissues. These findings suggest that HPMA copolymer conjugates have potential for targeted radiotherapy in cancer treatment [124]. Researchers developed a nanoscale drug delivery system using polymeric spherical nanoparticles labelled with _64_Cu and loaded with the antitumor drug docetaxel and the anti-inflammatory agent curcumin. The system was stabilized with Egg PG and PEGylated DSPE-PEG-COOH. PET/CT imaging was used to monitor the response to therapy. Results showed a synergistic effect when both drugs were delivered together, with 100% of mice surviving, compared to only 50% with DTX alone [125]. Polymeric nanostars functionalized with _89_Zr and _177_Lu were developed as a theranostic system for cancer. The nanostars showed higher accumulation in tumors and improved survival rates in mice, indicating their efficacy in passive targeting via the EPR effect [126]. Lactosomes, made of poly (L-lactic acid) and poly(sarcosine), were labelled with _18_F and used for PET imaging. The nanocarriers showed higher uptake in HeLa cells and successful in vivo tumor imaging using PET and NIRF labelling [127]. Researchers developed a new type of nanoparticle, called a polymeric unimolecular micelle, which can deliver chemotherapy and be used for MR imaging in cancer. They attached gadolinium (Gd) to the micelle’s surface using click chemistry. In vitro studies demonstrated higher T1 relaxivity on HeLa cells, indicating improved MR imaging. In vivo studies in rats showed increased accumulation in the liver and kidneys with positive contrast and longer circulation time [128].

## 14. Conclusions with Perspectives

The study of extracellular vesicles (ECVs), used as nanomedicine gadgets, has recently attracted the attention of a large number of researchers. Insight into ECV biology and behaviour may surely be gained by employing imaging modalities, presumably optical or radionuclide-based. This is plainly obvious from the research that has been done thus far. These techniques ought to be incorporated into facilitating their early development as possible treatments or diagnostic instruments. The radiolabelling techniques used and the results from SPECT/PET imaging were the main topics of this review. This research used intraluminal and membrane-based radiolabelling techniques, which fall into two primary types.

Our analysis highlights the significance of selecting the best radiolabelling technique for downstream application because each has benefits and drawbacks. This embraces the perks of incorporating in vivo ECV studies including envisioning with radionuclides, which have excellent sensitivity and quantification capabilities. This will help us better understand the fundamentals of these subgroups’ ECV biology, including their interactions with other cells and organs.

Additionally, the ability to evolve/optimize the strategy for foreseeable future clinical investigations is provided by the imaging modality’s availability in clinical settings. In this study, we have described numerous radiolabelling methods for biomedical applications of radioisotopes to ECVs. Several of the approaches are addressed, including covalent binding, intraluminal labelling, and membrane radiolabelling.

We highlight important elements such as radiolabelling effectiveness and stability in biological contexts since they are essential for an extensive depiction of how ECVs behave in real life throughout the course of their biological half-life. In the constantly developing field of nanotechnology, the approaches affirm how molecular envisioning with PET including SPECT can facilitate the ECVs biological creation for medical prognosis, including therapy. Exosomes hold great therapeutic potential, and numerous tracking techniques have been created to investigate their operation. Future research could use artificial intelligence, including machine learning, to scrutinize these data which determine whether the exosome movements indicate a low or high possibility of the therapy’s accomplishment quickly after treatment with exosomes is initiated, with the aid of the right imaging modalities.

Exosomes may be used to distribute drugs in a targeted manner in the future as more research results in a better knowledge of their biological functions. They would be able to treat particular patients with care for their unique needs while battling a variety of ailments. We will be able to create an expanded understanding of their capabilities and limitations with the help of an upcoming study, which is certain to occur.

## Figures and Tables

**Table 1:** Radiolabelling ECVs and their applications for drug delivery.

Source of Exosomes	Objective	Process	Outcomes	Ref.
Derived from MCF7 and MDA-MB-231 cells	In vivo biodistribution	Strain-promoted azide–alkyne click (SPAAC)	Enhanced distribution of the labelled exosomes	[39]
Macrophage-derived exosome	Biodistribution of ENVs in vivo	Incorporating 99mTc on the ECVs’ membrane surface	99mTc-HMPAO-ENVs accumulate in various organs	[25]
Erythrocyte-derived ECVs	Biodistribution of ECVs under SPECT/CT	Radiolabelling by 99mTc-tricarbonyl complexes with click chemistry	Aggregation of the 99mTc labelled ECVs in various organs	[40]
Milk-derived ECVs	Technique to get maximum ECV biodistribution	Radiochemical labelling by 99mTc (IV)	IV: decreased 99mTc-MDE aggregated; IP: decreased 99mTc-MDE distributed; IN: Biodistribution in the nasal cavity, trachea, and lungs	[41]
Mouse liver proliferative cell-derived ECVs	By glycosylation alteration of the biodistribution of ECVs	Labelled with ^124^I	Distributed primarily in the liver and lungs	[42]
Melanoma (B16F10)-derived ECVs	Novel, reliable, and universal radiolabelling approach	^111^Indium-chelated labelling of ECVs	Improved radiolabelling affectivity and radiochemical stability	[43]
Human umbilical cord cell-derived small ECVs	Using PET/MR	Surface modification of ECVs using ^64^Cu^2+^	Biodistribution in liver > lungs > kidney > stomach > brain (striatum, prefrontal cortex, and the cerebellum)	[44]
4T1 breast cancer-derived ECVs	Adequate imaging approach for ECVs for radioactivity quantification	Radiolabelled with a BFC-^64^Cu or ^68^Ga	Improved biodistribution of the BFC-_4_T1-ECVs	[45]
_4_T1 breast cancer-derived ECVs	Efficacy of PEGylation on ECVs	Radiolabelling of PEG conjugated ECVs	Resulted in enhanced pharmacokinetics of ECVs	[46]

**Table 2:** Radiolabelling of exosomes with SPECT and PET radioisotopes.

Type (SPECT Radioisotopes)	Radionuclide	RLY = Radiolabelling Yield	In Vitro Stability; Assessed by	In Vivo Imaging	Reference
Surface radiolabelling	125I-biotin	~80%	>95% serum stability at 4 h; UF	X	[52]
	Na131I + iodo-bead method	>80% for 4T1 ECVs	ca. 80% serum stability at 24 h; iTLC	✓	[53]
	99mTc-tricarbonyl	38.8 ± 6.2%	No data given	✓	[40]
	99mTc (+ SnCl2)	37 ± 9%	95% PBS stability at 48 h; iTLC	✓	[41]
	111In-DTPA	19.2 ± 4.5%	86.8 ± 3.1% PBS stability, and 80.4 ± 1.6% serum	✓	[43]
Intraluminal radiolabelling	111In-oxinate	81%	No data given	X	[54]
	111In-tropolone	4.7 ± 0.4%	43.4 ± 10.1% PBS stability, and 14.2 ± 2.8% serum stability at 24 h; SEC	✓	[43]
	99mTc-HMPAO	>93%	~90% serum stability at 5 h; iTLC	✓	[25]
	99mTc (+ SnCl2)	100%	93 ± 3% serum stability at 24 h; iTLC	✓	[55]
**Surface radiolabelling**	Na124I + iodogen method	Glycosylated = 17 ± 2%	>90% PBS stability at 72 h; iTLC	✓	[42]
	64Cu-DOTA	16–25%	94% serum stability at 24 h, 95% blood	✓	[44]
	64Cu-NOTA-Cy7	Non-PEGylated = 91.2 ± 0.2% PEGylated = 85.7 ± 0.7%	Non-PEGylated = 91.2 ± 0.2% PEGylated = 85.7 ± 0.7%	✓	[46]
	68Ga-NOTA-Cy7	Not reported	Not reported	Not reported	[45]
**Intraluminal radiolabelling**	89Zr-oxinate	6 ± 1%	Not reported	X	[56]
✓ = reported	X = not reported				

**Table 3:** Tracking of extracellular vesicles biodistribution using nuclear imaging strategy.

Labelling Method	Labelling Method	Radionuclide	ECVs (Markers)	In Vitro Stability	Ref.
Covalent binding	SPECT/CT	^99m^Tc-tricarbonyl complex	ECVs	Not reported	[4]
	PET	_124_I NaI	ECVs	NaCl	[42]
	SPECT/CT	_131_I	(CD9, CD63)	20% FBS	[53]
	SPECT/CT	^99m^Tc	Exosomes	PBS	[41]
Encapsulation	SPECT/CT	^99m^Tc-HMPAO	ENVs (CD63)	Serum or PBS	[25]
	Not reported	_111_In-oxine	Exosomes (HSP 70, 90, 27; CD9	Not reported	[54]
	SPECT/CT	_111_In via tropolone	Exosomes (CD81, CD9)	Serum or PBS	[43]
	Gamma camera	^99m^Tc	Exosome mimetics	PBS 20% FBS	[55]
Membrane radiolabelling	SPECT/CT	_111_In-DTPA	Exosomes (CD81, CD9)	50% FBS or PBS	[43]
	PET/MRI	_64_Cu-DOTA	ECVs (CD9, CD63, CD45)	PBS, serum	[44]
	PET	_64_Cu-NOTA-PEG	Exosomes	PBS or 25% mouse serum	[46]
	PET	_64_Cu-NOTA-PEG	Exosomes	_64_Cu-NOTA-PEG	[45]

**Table 4:** Brief description on labelling approach and in vivo tracking agent of ECVs.

Labelling Component	ECVs Source	Techniques Involved	Route of Administration	Ref.
DiR	Tissue explant	IVIS spectrum confocal microscopy	IV (Intravenous Route)	[97]
Cy5.5	Breast cancer cells	IVIS spectrum	IV Route	[39].
Enhanced GFP and tandem dimer Tomato	Mouse thymoma cell line	Multiphoton intravital microscopy	Endogenous generated ECVs	[90]
AIEgens (DPA-SCP)	Human placenta	IVIS spectrum confocal microscopy	IV Route	[98]
pHluorin	Yolk syncytial layer of zebra fish	Electron microscopy and fluorescent microscopy	Endogenous generated exosomes	[99]
Cre-loxP system with CFP, RFP, and GFP	Transplanted MDA-MB231 cells generated highly metastatic mammary tumors	Multi-photon high-resolution intravital imaging and confocal microscopy	Endogenous generated exosomes	[100]
CRISPR-Cas9 system with tdTomato	Tumor xenograft generated with transfected melanoma cells	Confocal microscopy	Endogenous generated exosomes	[101]
RLuc	CAL-62, MDA-MB-231 cells	IVIS spectrum	IV Route	[102]
ThermoLuc	HEK-293T cells	IVIS spectrum	IV Route	[103]
99mTc	HEK-293T transfected with HER2 target motif on the Lamp2B protein	Gamma camera	IV Route	[104]
^111^Indium	Melanoma cells	Gamma camera	IV Route	[102]
^64^Cu	hUCB-MNCs	Gamma camera and MRI scan	IV Route	[44]
Gold nanoparticles	Human MSCs	Micro-CT imaging	IV and IN (intranasal)	[95]
Quantum dots	HUVEC cells	IVIS spectrum	IT (intra-tumor injection.)	[105]

IVIS, in vivo imaging system; MSCs, mesenchymal stem cells; IV, intravenous injection; IN, intranasal administration; IT, intra-tumor injection.

## Data Availability

Data are contained within the article.

## References

[1] Théry C., Witwer K.W., Aikawa E., Alcaraz M.J., Anderson J.D., Andriantsitohaina R., Antoniou A., Arab T., Archer F., Atkin-Smith G.K. (2018). Minimal information for studies of extracellular vesicles 2018 (MISEV2018): A position statement of the International Society for Extracellular Vesicles and update of the MISEV2014 guidelines. J. Extracell. Vesicles.

[2] Maas S.L., Breakefield X.O., Weaver A.M. (2017). Extracellular vesicles: Unique intercellular delivery vehicles. Trends Cell Biol..

[3] Xiao Y., Zheng L., Zou X., Wang J., Zhong J., Zhong T. (2019). Extracellular vesicles in type 2 diabetes mellitus: Key roles in pathogenesis, complications, and therapy. J. Extracell. Vesicles.

[4] Oggero S., Austin-Williams S., Norling L.V. (2019). The contrasting role of extracellular vesicles in vascular inflammation and tissue repair. Front. Pharmacol..

[5] Murphy D.E., de Jong O.G., Brouwer M., Wood M.J., Lavieu G., Schiffelers R.M., Vader P. (2019). Extracellular vesicle-based therapeutics: Natural versus engineered targeting and trafficking. Exp. Mol. Med..

[6] Simeone P., Bologna G., Lanuti P., Pierdomenico L., Guagnano M.T., Pieragostino D., Del Boccio P., Vergara D., Marchisio M., Miscia S. (2020). Extracellular Vesicles as Signaling Mediators and Disease Biomarkers across Biological Barriers. Int. J. Mol. Sci..

[7] Yang B., Chen Y., Shi J. (2019). Exosome biochemistry and advanced nanotechnology for next-generation theranostic platforms. Adv. Mater..

[8] Lane R., Korbie D., Hill M., Trau M. (2018). Extracellular vesicles as circulating cancer biomarkers: Opportunities and challenges. Clin. Transl. Med..

[9] Lorenc T., Chrzanowski J., Olejarz W. (2020). Current Perspectives on Clinical Use of Exosomes as a Personalized Contrast Media and Theranostics. Cancers.

[10] Luan X., Sansanaphongpricha K., Myers I., Chen H., Yuan H., Sun D. (2017). Engineering exosomes as refined biological nanoplatforms for drug delivery. Acta Pharmacol. Sin..

[11] Zheng Y., Hasan A., Babadaei M.M.N., Behzadi E., Nouri M., Sharifi M., Falahati M. (2020). Exosomes: Multiple-targeted multifunctional biological nanoparticles in the diagnosis, drug delivery, and imaging of cancer cells. Biomed. Pharmacother..

[12] Di Rocco G., Baldari S., Toietta G. (2016). Towards Therapeutic Delivery of Extracellular Vesicles: Strategies for In Vivo Tracking and Biodistribution Analysis. Stem Cells Int..

[13] Adem B., Melo S.A. (2017). Animal models in exosomes research: What the future holds. Nov. Implic. Exosomes Diagn. Treat. Cancer Infect. Dis..

[14] Wu M., Shu J. (2018). Multimodal molecular imaging: Current status and future directions. Contrast Media Mol. Imaging.

[15] Kim D.H., Kothandan V.K., Kim H.W., Kim K.S., Kim J.Y., Cho H.J., Lee Y.-K., Lee D.-E., Hwang S.R. (2019). Noninvasive Assessment of Exosome Pharmacokinetics in Vivo: A Review. Pharmaceutics.

[16] Patra J.K., Das G., Fraceto L.F., Campos E.V.R., del Pilar Rodriguez-Torres M., Acosta-Torres L.S., Diaz-Torres L.A., Grillo R., Swamy M.K., Sharma S. (2018). Nano based drug delivery systems: Recent developments and future prospects. J. Nanobiotechnol..

[17] Mitchell M.J., Billingsley M.M., Haley R.M., Wechsler M.E., Peppas N.A., Langer R. (2020). Engineering precision nanoparticles for drug delivery. Nat. Rev. Drug Discov..

[18] Tyagi A., Sharma P.K., Malviya R. (2018). Role of blood retinal barrier in drug absorption. Pharm. Anal. Acta.

[19] Anselmo A., Gupta V., Zern B.J., Pan D., Zakrewsky M., Muzykantov V., Mitragotri S. (2013). Delivering Nanoparticles to Lungs while Avoiding Liver and Spleen through Adsorption on Red Blood Cells. ACS Nano.

[20] De Jong W.H., Borm P.J. (2008). Drug delivery and nanoparticles: Applications and hazards. Int. J. Nanomed..

[21] Luo R., Liu M., Tan T., Yang Q., Wang Y., Men L., Zhao L., Zhang H., Wang S., Xie T. (2021). Emerging Significance and Therapeutic Potential of Extracellular vesicles. Int. J. Biol. Sci..

[22] Jansen F., Li Q., Pfeifer A., Werner N. (2017). Endothelial- and Immune Cell-Derived Extracellular Vesicles in the Regulation of Cardiovascular Health and Disease. JACC Basic Transl. Sci..

[23] Dang X.T., Kavishka J.M., Zhang D.X., Pirisinu M., Le M.T. (2020). Extracellular vesicles as an efficient and versatile system for drug delivery. Cells.

[24] Sork H., Corso G., Krjutskov K., Johansson H.J., Nordin J., Wiklander O.P.B., Lee Y.X.F., Westholm J.O., Lehtiö J., Wood M.J.A. (2018). Heterogeneity and interplay of the extracellular vesicle small RNA transcriptome and proteome. Sci. Rep..

[25] Hwang D.W., Choi H., Jang S.C., Yoo M.Y., Park J.Y., Choi N.E., Oh H.J., Ha S., Lee Y.S., Jeong J.M. (2015). Noninvasive imaging of radiolabeled exosome-mimetic nanovesicle using 99mTc-HMPAO. Sci. Rep..

[26] Ullal A.J., Pisetsky D.S., Reich C.F. (2010). Use of SYTO 13, a fluorescent dye binding nucleic acids, for the detection of microparticles in in vitro systems. Cytometry Part A: The Journal of the International Society. Adv. Cytom..

[27] Syn N.L., Wang L., Chow E.K., Lim C.T., Goh B.C. (2017). Exosomes in Cancer Nanomedicine and Immunotherapy: Prospects and Challenges. Trends Biotechnol..

[28] Hwang D.W. (2019). Perspective in Nuclear Theranostics Using Exosome for the Brain. Nucl. Med. Mol. Imaging.

[29] Peer D., Karp J.M., Hong S., Farokhzad O.C., Margalit R., Langer R. (2007). Nanocarriers as an emerging platform for cancer therapy. Nat. Nanotech..

[30] Berenguer J., Lagerweij T., Zhao X.W., Dusoswa S., Van Der Stoop P., Westerman B., Gooijer M.C., Zoetemelk M., Zomer A., Crommentuijn M.H. (2018). Glycosylated extracellular vesicles released by glioblastoma cells are decorated by CCL18 allowing for cellular uptake via chemokine receptor CCR8. J. Extracell. Vesicles.

[31] Rana S., Yue S., Stadel D., Zoller M. (2012). Toward tailored exosomes: The exosomal tetraspanin web contributes to target cell selection. Int. J. Biochem. Cell Biol..

[32] Walker S., Busatto S., Pham A., Tian M., Suh A., Carson K. (2019). Extracellular vesicle-based drug delivery systems for cancer treatment. Theranostics.

[33] Lai C.P., Tannous B.A., Breakefield X.O. (2014). Noninvasive in vivo monitoring of extracellular vesicles. Bioluminescent Imaging: Methods and Protocols. Methods Mol. Biol..

[34] Alvarez-Erviti L., Seow Y., Yin H., Betts C., Lakhal S., Wood M.J. (2011). Delivery of siRNA to the mouse brain by systemic injection of targeted exosomes. Nat. Biotechnol..

[35] Chuo S.T., Chien J.C., Lai C.P. (2018). Imaging extracellular vesicles: Current and emerging methods. J. Biomed. Sci..

[36] Rahmim A., Zaidi H. (2008). PET versus SPECT: Strengths, limitations and challenges. Nucl. Med. Commun..

[37] Antimisiaris S.G., Mourtas S., Marazioti A. (2018). Exosomes and Exosome-Inspired Vesicles for Targeted Drug Delivery. Pharmaceutics.

[38] Man F., Gawne P.J., de Rosales R.T.M. (2019). Nuclear imaging of liposomal drug delivery systems: A critical review of radiolabelling methods and applications in nanomedicine. Adv. Drug Deliv. Rev..

[39] Lee T.S., Kim Y., Zhang W., Song I.H., Tung C.-H. (2018). Facile metabolic glycan labeling strategy for exosome tracking. Biochim. Biophys. Acta Gen. Subj..

[40] Varga Z., Gyurkó I., Pálóczi K., Buzás E.I., Horváth I., Hegedűs N., Máthé D., Szigeti K. (2016). Radiolabeling of extracellular vesicles with 99mTc for quantitative in vivo imaging studies. Cancer Biother. Radiopharm..

[41] González M.I., Martín-Duque P., Desco M., Salinas B. (2020). Radioactive Labeling of Milk-Derived Exosomes with 99mTc and In Vivo Tracking by SPECT Imaging. Nanomaterials.

[42] Royo F., Cossío U., Ruiz de Angulo A., Llop J., Falcon-Perez J.M. (2019). Modification of the glycolsylation of extracellular vesicles alters their biodistribution in mice. Nanoscale.

[43] Faruqu F.N., Wang J.T., Xu L., McNickle L., Chong E.M., Walters A., Gurney M., Clayton A., Smyth L.A., Hider R. (2019). Membrane radiolabelling of exosomes for comparative biodistribution analysis in immunocompetent and immunodeficient mice-a novel and universal approach. Theranostics.

[44] Banerjee A., Alves V., Rondão T., Sereno J., Neves Â., Lino M., Ribeiro A., Abrunhosa A.J., Ferreira L.S. (2019). A positron-emission tomography (PET)/magnetic resonance imaging (MRI) platform to track in vivo small extracellular vesicles. Nanoscale.

[45] Jung K.O., Kim Y.H., Chung S.J., Kang K.W., Rhee S., Pratx G., Chung J.K., Youn H. (2020). Highly sensitive identification of lymphatic and Hematogenous metastasis routes of novel radiolabeled exosomes using non-invasive PET imaging. bioRxiv.

[46] Shi S., Li T., Wen X., Wu S.Y., Xiong C., Zhao J., Lincha V.R., Chow D.S., Liu Y., Sood A.K. (2019). Copper-64 labeled PEGylated exosomes for in vivo positron emission tomography and enhanced tumor retention. Bioconjug. Chem..

[47] Hoshino A., Costa-Silva B., Shen T.-L., Rodrigues G., Hashimoto A., Mark M.T., Molina H., Kohsaka S., Di Giannatale A., Ceder S. (2015). Tumour exosome integrins determine organotropic metastasis. Nature.

[48] Rayamajhi S., Aryal S. (2020). Surface functionalization strategies of extracellular vesicles. J. Mater. Chem. B.

[49] Almeida S., Santos L., Falcão A., Gomes C., Abrunhosa A. (2020). In vivo tracking of extracellu-lar vesicles by nuclear imaging: Advances in radiolabeling strategies. Int. J. Mol. Sci..

[50] Phillips W.T., Rudolph A.S., Goins B., Timmons J.H., Klipper R., Blumhardt R. (1992). A simple method for producing a technetium-99m-labeled liposome which is stable In Vivo. Int. J. Radiat. Appl. Instrum. B.

[51] Edmonds S., Volpe A., Shmeeda H., Parente-Pereira A.C., Radia R., Baguna-Torres J., Szanda I., Severin G.W., Livieratos L., Blower P.J. (2016). Exploiting the Metal-Chelating Properties of the Drug Cargo for In Vivo Positron Emission Tomography Imaging of Liposomal Nanomedicines. ACS Nano.

[52] Morishita M., Takahashi Y., Nishikawa M., Sano K., Kato K., Yamashita T., Imai T., Saji H., Takakura Y. (2015). Quantitative analysis of tissue distribution of the B16BL6-derived exosomes using a streptavidin-lactadherin fusion protein and Iodine-125-Labelled biotin derivative after intravenous injection in mice. J. Pharm. Sci..

[53] Rashid M.H., Borin T.F., Ara R., Angara K., Cai J., Achyut B.R., Liu Y., Arbab A.S. (2019). Differential in vivo biodistribution of 131I-labeled exosomes from diverse cellular origins and its implication for theranostic application. Nanomedicine.

[54] Smyth T., Kullberg M., Malik N., Smith-Jones P., Graner M.W., Anchordoquy T.J. (2015). Biodistribution and delivery efficiency of unmodified tumor-derived exosomes. J. Control. Release.

[55] Gangadaran P., Hong C.M., Oh J.M., Rajendran R.L., Kalimuthu S., Son S.H., Gopal A., Zhu L., Baek S.H., Jeong S.Y. (2018). In vivo non-invasive imaging of radio-labeled exosome-mimetics derived from red blood cells in mice. Front. Pharmacol..

[56] Khan A.A., Man F., Faruqu F.N., Kim J., Al-Salemee F., Carrascal-Miniño A., Volpe A., Liam-Or R., Simpson P., Fruhwirth G.O. (2022). PET imaging of small extracellular vesicles via [89Zr] zr (oxinate) 4 direct radiolabeling. Bioconjug. Chem..

[57] Jang S.C., Kim O.Y., Yoon C.M., Choi D.S., Roh T.Y., Park J., Nilsson J., Lotvall J., Kim Y.K., Gho Y.S. (2013). Bioinspired exosome-mimetic nanovesicles for targeted delivery of chemotherapeutics to malignant tumors. ACS Nano.

[58] Son S.H., Oh J.M., Gangadaran P., Ji H.D., Lee H.W., Rajendran R.L., Baek S.H., Gopal A., Kalimuthu S., Jeong S.Y. (2019). White blood cell labeling with Technetium-99m (99mTc) using red blood cell extracellular vesicles-mimetics. Blood Cells Mol. Dis..

[59] Betzer O., Barnoy E., Sadan T., Elbaz I., Braverman C., Liu Z., Popovtzer R. (2020). Advances in imaging strategies for in vivo tracking of exosomes. Wiley Interdiscip. Rev. Nanomed. Nanobiotech..

[60] Sancho-Albero M., Ayaz N., Sebastian V., Chirizzi C., Encinas-Gimenez M., Neri G., Chaabane L., Luján L., Martin-Duque P., Metrangolo P. (2023). Superfluorinated Extracellular Vesicles for In Vivo Imaging by 19F-MRI. ACS Appl. Mater. Interfaces.

[61] Wester H.J. (2007). Nuclear imaging probes: From bench to bedside. Clin. Cancer Res..

[62] Li Y.J., Wu J.Y., Wang J.M., Hu X.B., Xiang D.X. (2020). Emerging strategies for labeling and tracking of extracellular vesicles. J. Control. Release.

[63] Clayton A., Harris C.L., Court J., Mason M.D., Morgan B.P. (2003). Antigen-presenting cell exosomes are protected from complement-mediated lysis by expression of CD55 and CD59. Eur. J. Immunol..

[64] Saunderson S.C., Dunn A.C., Crocker P.R., McLellan A.D. (2014). CD169 mediates the capture of exosomes in the spleen and lymph nodes. Blood.

[65] Choi H.S., Liu W., Misra P., Tanaka E., Zimmer J.P., Ipe B.I., Bawendi M.G., Frangioni J.V. (2007). Renal clearance of quantum dots. Nat. Biotech..

[66] Lobo E.D., Hansen R.J., Balthasar J.P. (2004). Antibody Pharmacokinetics and Pharmacodynamics. J. Pharm. Sci..

[67] Nair D.P., Podgorski M., Chatani S., Gong T., Xi W., Fenoli C.R., Bowman C.N. (2014). The thiol-Michael addition click reaction: A powerful and widely used tool in materials chemistry. Chem. Mater..

[68] Dalton H.D., Cocks A., Falcon-Perez J.M., Sayers E.J., Webber J.P., Watson P., Clayton A., Jones A.T. (2017). Fluorescence labelling of extracellular vesicles using a novel thiol-based strategy for quantitative analysis of cellular delivery and intracellular traffic. Nanoscale.

[69] Fan Z., Xiao K., Lin J., Liao Y., Huang X. (2019). Functionalized DNA enables programming exosomes/vesicles for tumor imaging and therapy. Small.

[70] Han Q., Xie Q.R., Li F., Cheng Y., Wu T., Zhang Y., Lu X., Wong A.S., Sha J., Xia W. (2021). Targeted inhibition of SIRT6 via engineered exosomes impairs tumorigenesis and metastasis in prostate cancer. Theranostics.

[71] Hong C.S., Funk S., Muller L., Boyiadzis M., Whiteside T.L. (2016). Isolation of biologically ac-tive and morphologically intact exosomes from plasma of patients with cancer. J. Extracell. Vesicles.

[72] Mori K., Hirase M., Morishige T., Takano E., Sunayama H., Kitayama Y., Inubushi S., Sasaki R., Yashiro M., Takeuchi T. (2019). A Pretreatment-Free, Polymer-Based Platform Prepared by Molecular Imprinting and Post-Imprinting Modifications for Sensing Intact Exosomes. Angew. Chem..

[73] Wiklander O.P., Nordin J.Z., O’Loughlin A., Gustafsson Y., Corso G., Mäger I., Vader P., Lee Y., Sork H., Seow Y. (2015). Extracellular vesicle in vivo biodistribution is determined by cell source, route of administration and targeting. J. Extracell. Vesicles.

[74] Peinado H., Alečković M., Lavotshkin S., Matei I., Costa-Silva B., Moreno-Bueno G., Hergueta-Redondo M., Williams C., García-Santos G., Ghajar C.M. (2012). Melanoma exosomes educate bone marrow progenitor cells toward a pro-metastatic phenotype through MET. Nat. Med..

[75] Grange C., Tapparo M., Bruno S., Chatterjee D., Quesenberry P.J., Tetta C., Camussi G. (2014). Biodistribution of mesenchymal stem cell-derived extracellular vesicles in a model of acute kidney injury monitored by optical imaging. Int. J. Mol. Med..

[76] Saari H., Lazaro-Ibanez E., Viitala T., Vuorimaa-Laukkanen E., Siljander P., Yliperttula M. (2015). Microvesicle- and exosome-mediated drug delivery enhances the cytotoxicity of Paclitaxel in autologous prostate cancer cells. J Control. Release.

[77] Suetsugu A., Honma k., Saji S., Moriwaki H., Ochiya T., Hoffman R.M. (2013). Imaging exosome transfer from breast cancer cells to stroma at metastatic sites in orthotopic nude-mouse models. Adv. Drug Deliv. Rev..

[78] Takahashi Y., Nishikawa M., Shinotsuka H., Matsui Y., Ohara S., Imai T., Takakura Y. (2013). Visual-ization and in vivo tracking of the exosomes of murine melanoma B16-BL6 cells in mice after intravenous injection. J. Biotech..

[79] Hu L., Wickline S.A., Hood J.L. (2014). Magnetic resonance imaging of melanoma exosomes in lymph nodes. Magn. Reson. Med..

[80] Dabrowska S., Del Fattore A., Karnas E., Frontczak-Baniewicz M., Kozlowska H., Muraca M., Janowski M., Lukomska B. (2018). Imaging of extracellular vesicles derived from human bone marrow mesenchymal stem cells using fluorescent and magnetic labels. Int. J. Nanomed..

[81] Liu T., Zhu Y., Zhao R., Wei X., Xin X. (2020). Visualization of exosomes from mesenchymal stem cells in vivo by magnetic resonance imaging. Magn. Reson. Imaging.

[82] Chen X., Lan J., Liu Y., Li L., Yan L., Xia Y., Wu F., Li C., Li S., Chen J. (2018). A paper-supported aptasensor based on upconversion luminescence resonance energy transfer for the accessible determination of exosomes. Biosens. Bioelectron..

[83] Qiao B., Guo Q., Jiang J., Qi Y., Zhang H., He B., Cai C., Shen J. (2019). An electrochemilumi-nescent aptasensor for amplified detection of exosomes from breast tumor cells (MCF-7 cells) based on G-quadruplex/hemin DNAzymes. Analyst.

[84] Fang D., Zhao D., Zhang S., Huang Y., Dai H., Lin Y. (2020). Black phosphorus quantum dots functionalized MXenes as the enhanced dual-mode probe for exosomes sensing. Sens. Actuators B Chem..

[85] Yu Q., Zhao Q., Wang S., Zhao S., Zhang S., Yin Y., Dong Y. (2020). Development of a lateral flow aptamer assay strip for facile identification of theranostic exosomes isolated from human lung carcinoma cells. Anal. Biochem..

[86] Wang Z.L., Zong S.F., Wang Y.J., Li N., Li L., Lu J., Wang Z.Y., Chen B.A., Cui Y.P. (2018). Screening and multiple detection of cancer exosomes using an SERS-based method. Nanoscale.

[87] Wang Q., Zou L., Yang X., Liu X., Nie W., Zheng Y., Cheng Q., Wang K. (2019). Direct quan-tification of cancerous exosomes via surface plasmon resonance with dual gold nanoparticle-assisted signal amplification. Biosens. Bioelectron..

[88] Zhu F., Li D., Ding Q., Lei C., Ren L., Ding X., Sun X. (2019). 2D magnetic MoS_2_-Fe_3_O_4_ hybrid nanostructures for ultrasensitive exosome detection in GMR sensor. Biosens. Bioelectron..

[89] Shen L.M., Quan L., Liu J. (2018). Tracking exosomes in vitro and in vivo to elucidate their phys-iological functions: Implications for diagnostic and therapeutic nanocarriers. ACS Appl. Nano Mater..

[90] Lai C.P., Kim E.Y., Badr C.E., Weissleder R., Mempel T.R., Tannous B.A., Breakefield X.O. (2015). Visualization and tracking of tumour extracellular vesicle delivery and RNA translation using multiplexed reporters. Nat. Commun..

[91] Chen C., Zong S., Wang Z., Lu J., Zhu D., Zhang Y., Cui Y. (2016). Imaging and intracellular tracking of cancer-derived exosomes using single-molecule localization-based super-resolution microscope. ACS Appl. Mater. Interfaces.

[92] Jiang X., Zong S., Chen C., Zhang Y., Wang Z., Cui Y. (2018). Gold–carbon dots for the intracellular imaging of cancer-derived exosomes. Nanotechnology.

[93] Gupta D., Liang X., Pavlova S., Wiklander O.P., Corso G., Zhao Y., Saher O., Bost J., Zickler A.M., Piffko A. (2020). Quantification of extracellular vesicles in vitro and in vivo using sensitive bioluminescence imaging. J. Extracell. Vesicles.

[94] Zhuang M., Chen X., Du D., Shi J., Deng M., Long Q., Yin X., Wang Y., Rao L. (2020). SPION decorated exosome delivery of TNF-α to cancer cell membranes through magnetism. Nanoscale.

[95] Betzer O., Perets N., Angel A., Motiei M., Sadan T., Yadid G., Offen D., Popovtzer R. (2017). In vivo neuroimaging of exosomes using gold nanoparticles. ACS Nano.

[96] Lázaro-Ibáñez E., Faruqu F.N., Saleh A.F., Silva A.M., Wang J.T.-W., Rak J., Al-Jamal K.T., Dekker N. (2021). Selection of fluorescent, bioluminescent, and radioactive tracers to accurately reflect extracellular vesicle biodistribution in vivo. ACS Nano.

[97] Sun W., Li Z., Zhou X., Yang G., Yuan L. (2019). Efficient exosome delivery in refractory tissues assisted by ultrasound-targeted microbubble destruction. Drug. Deliv..

[98] Cao H., Yue Z., Gao H., Chen C., Cui K., Zhang K., Cheng Y., Shao G., Kong D., Li Z. (2019). In vivo real-time imaging of extracellular vesicles in liver regeneration via aggregation-induced emission luminogens. ACS Nano.

[99] Verweij F.J., Revenu C., Arras G., Dingli F., Loew D., Pegtel D.M., Follain G., Allio G., Goetz J.G., Zimmermann P. (2019). Live tracking of inter-organ communication by endogenous exosomes in vivo. Dev. Cell.

[100] Zomer A., Steenbeek S.C., Maynard C., Van Rheenen J. (2016). Studying extracellular vesicle transfer by a Cre-loxP method. Nat. Protoc..

[101] Ye Y., Shi Q., Yang T., Xie F., Zhang X., Xu B., Fang J., Chen J., Zhang Y., Li J. (2022). In vivo visualized tracking of tumor-derived extracellular vesicles using CRISPR-cas9 system. Technol. Cancer. Res. Treat..

[102] Gangadaran P., Li X.J., Lee H.W., Oh J.M., Kalimuthu S., Rajendran R.L., Son S.H., Baek S.H., Singh T.D., Zhu L. (2017). A new bioluminescent reporter system to study the biodistribution of systematically injected tumor-derived bioluminescent extracellular vesicles in mice. Oncotarget.

[103] Wang C., Li N., Li Y., Hou S., Zhang W., Meng Z., Wang S., Jia Q., Tan J., Wang R. (2022). Engineering a HEK-293T exosome-based delivery platform for efficient tumor-targeting chemotherapy/internal irradiation combination therapy. J. Nanobiotech..

[104] Molavipordanjani S., Khodashenas S., Abedi S.M., Moghadam M.F., Mardanshahi A., Hosseinimehr S.J. (2020). 99mTc-radiolabeled HER2 targeted exosome for tumor imaging. Eur. J. Pharm. Sci..

[105] Chen G., Zhu J.Y., Zhang Z.L., Zhang W., Ren J.G., Wu M., Hong Z.Y., Lv C., Pang D.W., Zhao Y.F. (2015). Transformation of cell-derived microparticles into quantum-dot-labeled nanovectors for antitumor siRNA delivery. Angew. Chem..

[106] Pellico J., Ruiz-Cabello J., Saiz-Alía M., del Rosario G., Caja S., Montoya M., de Manuel L.F., Morales M.P., Gutiérrez L., Galiana B. (2016). Fast synthesis and bioconjugation of 68Ga core-doped extremely small iron oxide nanoparticles for PET/MR imaging. Contrast Media Mol. Imaging.

[107] Koziorowski J., Stanciu A.E., Gomez-Vallejo V., Llop J. (2017). Radiolabeled nanoparticles for cancer diagnosis and therapy. Curr. Med. Chem. Anticancer Agents.

[108] Same S., Aghanejad A., Nakhjavani S.A., Barar J., Omidi Y. (2016). Radiolabeled theranostics: Magnetic and gold nanoparticles. Bioimpacts.

[109] Lee S.B., Ahn S.B., Lee S.W., Jeong S.Y., Ghilsuk Y., Ahn B.C., Kim E.M., Jeong H.J., Lee J., Lim D.K. (2016). Radionuclide-embedded gold nanoparticles for enhanced dendritic cell-based cancer immunotherapy, sensitive and quantitative tracking of dendritic cells with PET and Cerenkov luminescence. NPG Asia Mater..

[110] Kao H.W., Lin Y.Y., Chen C.C., Chi K.H., Tien D.C., Hsia C.C., Lin M.H., Wang H.E. (2013). Evaluation of EGFR-targeted radioimmuno-gold-nanoparticles as a theranostic agent in a tumor animal model. Bioorg. Med. Chem. Lett..

[111] Li X., Wang C., Tan H., Cheng L., Liu G., Yang Y., Zhao Y., Zhang Y., Li Y., Zhang C. (2016). Gold nanoparticles-based SPECT/CT imaging probe targeting for vulnerable atherosclerosis plaques. Biomaterials.

[112] Zhao Y., Detering L., Sultan D., Cooper M.L., You M., Cho S., Meier S.L., Luehmann H., Sun G., Rettig M. (2016). Gold nanoclusters doped with 64Cu for CXCR4 positron emission tomography imaging of breast cancer and metastasis. ACS Nano.

[113] El-Ghareb W.I., Swidan M.M., Ibrahim I.T., Abd El-Bary A., Tadros M.I., Sakr T.M. (2020). 99mTc-Doxorubicin-loaded gallic acid-gold nanoparticles ( 99mTc-DOX-loaded GAAu NPs) as a multifunctional theranostic agent. Int. J. Pharm..

[114] England C.G., Im H.-J., Feng L., Chen F., Graves S.A., Hernandez R., Orbay H., Xu C., Cho S.Y., Nickles R.J. (2016). Re-assessing the enhanced permeability and retention effect in peripheral arterial disease using radiolabeled long circulating nanoparticles. Biomaterials.

[115] Mokhodoeva O., Vlk M., Málková E., Kukleva E., Mičolová P., Štamberg K., Šlouf M., Dzhenloda R., Kozempel J. (2016). Study of 223Ra uptake mechanism by Fe3O4 nanoparticles: Towards new prospective theranostic SPIONs. J. Nanoparticle Res..

[116] Chakravarty R., Valdovinos H.F., Chen F., Lewis C.M., Ellison P.A., Luo H., Meyerand M.E., Nickles R.J., Cai W. (2014). Intrinsically germanium-69-labeled iron oxide nanoparticles: Synthesis and in-vivo dual-modality PET/MR imaging. Adv. Mater. Res..

[117] Su T., Wang Y.B., Han D., Wang J., Qi S., Gao L., Shao Y.H., Qiao H.Y., Chen J.W., Liang S.H. (2017). Multimodality imaging of angiogenesis in a rabbit atherosclerotic model by GEBP11 peptide targeted nanoparticles. Theranostics.

[118] Rosenholm J.M., Mamaeva V., Sahlgren C., Lindén M. (2012). Nanoparticles in targeted cancer therapy: Mesoporous silica nanoparticles entering preclinical development stage. Nanomedicine.

[119] Yang Y., Liu Y., Cheng C., Shi H., Yang H., Yuan H., Ni C. (2017). Rational design of GOmodified Fe_3_O_4_/SiO_2_ nanoparticles with combined rhenium-188 and gambogic acid for magnetic target therapy. ACS Appl. Mater. Interfaces.

[120] Gao H., Liu X., Tang W., Niu D., Zhou B., Zhang H., Liu W., Gu B., Zhou X., Zheng Y. (2016). 99m Tc-conjugated manganese-based mesoporous silica nanoparticles for SPECT, pH-responsive MRI and anti-cancer drug delivery. Nanoscale.

[121] Yamaguchi H., Tsuchimochi M., Hayama K., Kawase T., Tsubokawa N. (2016). Duallabeled near-infrared/99mTc imaging probes using PAMAM-coated silica nanoparticles for the imaging of HER2-expressing cancer cells. Int. J. Mol. Sci..

[122] Lammers T.G., Subr V., Peschke P., Kühnlein R., Hennink W.E., Ulbrich K., Kiessling F., Heilmann M., Debus J., Huber P.E. (2008). Image-guided and passively tumour-targeted polymeric nanomedicines for radiochemotherapy. Br. J. Cancer.

[123] Koziolova E., Goel S., Chytil P., Janoušková O., Barnhart T.E., Cai W., Etrych T. (2017). A tumor-targeted polymer theranostics platform for positron emission tomography and fluorescence imaging. Nanoscale.

[124] Mitra A., Nan A., Papadimitriou J.C., Ghandehari H., Line B.R. (2006). Polymer-peptide conju-gates for angiogenesis targeted tumor radiotherapy. Nucl. Med. Biol..

[125] Stigliano C., Key J., Ramirez M., Aryal S., Decuzzi P. (2015). Radiolabeled polymeric nanoconstructs loaded with docetaxel and curcumin for cancer combinatorial therapy and nuclear imaging. Adv. Funct. Mater..

[126] Goos J.A., Cho A., Carter L.M., Dilling T.R., Davydova M., Mandleywala K., Puttick S., Gupta A., Price W.S., Quinn J.F. (2020). Delivery of polymeric nanostars for molecular imaging and endoradiotherapy through the enhanced permeability and retention (EPR) effect. Theranostics.

[127] Yamamoto F., Yamahara R., Makino A., Kurihara K., Tsukada H., Hara E., Hara I., Kizaka-Kondoh S., Ohkubo Y., Ozeki E. (2013). Radiosynthesis and initial evaluation of 18F labeled nanocarrier composed of poly (L-lactic acid)-block-poly (sarcosine) amphiphilic polydepsipeptide. Nucl. Med. Biol..

[128] Li X., Qian Y., Liu T., Hu X., Zhang G., You Y., Liu S. (2011). Amphiphilic multiarm star block copolymer-based multifunctional unimolecular micelles for cancer targeted drug delivery and MR imaging. Biomaterials.

